# Molecular Pathogenesis of Glioblastoma in Adults and Future Perspectives: A Systematic Review

**DOI:** 10.3390/ijms23052607

**Published:** 2022-02-26

**Authors:** Yagmur Esemen, Mariam Awan, Rabeeia Parwez, Arsalan Baig, Shahinur Rahman, Ilaria Masala, Sonia Franchini, Dimitrios Giakoumettis

**Affiliations:** 1 Neurosurgical Department, Queen’s Hospital, Romford, London RM7 0AG, UK; yagmur.esemen@nhs.net (Y.E.); m.awan1@nhs.net (M.A.); rabeeia.parwez@nhs.net (R.P.); arsalan.baig@nhs.net (A.B.); rahman.shahinur@nhs.net (S.R.); 2 Department of Trauma and Orthopedics, The James Cook University Hospital, Middlesbrough TS4 3BW, UK; masalailaria09@gmail.com; 3 General Surgery Department, Queen’s Hospital, Romford, London RM7 0AG, UK; sonia.franchini@nhs.net

**Keywords:** glioblastoma, molecular pathogenesis, signaling pathways, gliomagenesis, theranostics, vaccines

## Abstract

Glioblastoma (GBM) is the most common and malignant tumour of the central nervous system. Recent appreciation of the heterogeneity amongst these tumours not only changed the WHO classification approach, but also created the need for developing novel and personalised therapies. This systematic review aims to highlight recent advancements in understanding the molecular pathogenesis of the GBM and discuss related novel treatment targets. A systematic search of the literature in the PubMed library was performed following the PRISMA guidelines for molecular pathogenesis and therapeutic advances. Original and meta-analyses studies from the last ten years were reviewed using pre-determined search terms. The results included articles relevant to GBM development focusing on the aberrancy in cell signaling pathways and intracellular events. Theragnostic targets and vaccination to treat GBM were also explored. The molecular pathophysiology of GBM is complex. Our systematic review suggests targeting therapy at the stemness, p53 mediated pathways and immune modulation. Exciting novel immune therapy involving dendritic cell vaccines, B-cell vaccines and viral vectors may be the future of treating GBM.

## 1. Introduction

Glioma is an umbrella term used to describe primary brain tumours. Glioblastoma Multiforme (GBM) is the commonest glioma in adults, and it accounts for more than 60% of all adult brain tumours [1]. Traditionally, the classification of gliomas was based on its presumed cell of origin and histological features of malignancy. More recently, the updated World Health Organization (WHO) classification took a new approach by integrating molecular markers in the diagnostic process [2]. Glioblastoma remains a significant public health burden. International incidence of glioma is reported as 5.3 per 100,000 population per year and seems to be higher in the Western world [3]. Incidence peaks between ages 55 to 60 [4], and the prevalence is higher in males, with a relative sex ratio of 1.66 in England and 1.56 in the USA. Median survival for GBM has been reported as 6.1 months with 1, 2 and 5-year survivals estimated as 28.4%, 11.5% and 3.4%, respectively [5]. Treatment of GBM has been evolving. Traditionally, GBM treatment included surgery followed by radiotherapy, where the 3- and 5-year survival rates were 4.4 and 1.9%, respectively [6]. In 2005, the addition of Temozolomide (TMZ), an oral alkylating chemotherapy agent, showed significant improvement in overall survival where 3- and 5-year survival rates rose to 16% and 9.8%, respectively [7]. This led to the development of the “Stupp Protocol”, where combining TMZ with radiotherapy has been accepted as the “standard therapy” for GBM worldwide [8]. Even with these advancements, GBM remains challenging to treat. The new WHO classification of gliomas highlights the importance of molecular markers in diagnosis. Current therapy of GBM seems to have reached its limits and cannot address the genetic diversity and multiplicity of the tumor. Therefore, in order to treat GBM, we first need to understand it better. This systematic review will focus on new research regarding gliomagenesis and novel therapeutics of glioblastoma multiforme.

### Background

Glioblastoma multiforme remains a notoriously hard to treat cancer associated with treatment resistance and high recurrence rates. A great deal of effort has been invested in understanding GBM pathophysiology to address the challenges in treatment. Gliomagenesis, the formation and development of gliomas, is a multistep process in which normal cells undergo genetic alterations that lead to malignant derivatives [9]. Cells progressively acquire genetic and epigenetic changes resulting in inhibiting tumour suppressor genes (TSG) and activation of proto-oncogenes. Together, these changes help cells escape the body’s regulatory mechanisms and lead to rapidly proliferating, undifferentiated cell clusters, known as tumours. These tumours become malignant when they invade and migrate into other tissues.

At a cellular level, certain tumorigenic processes have been associated with the development and progression of GBMs. These intracellular events include but are not limited to loss of cell cycle control, growth factor over-expression, angiogenesis, invasion & migration, genetic instability and disorder of apoptosis [10]. Complexity of GBMs arises at the cellular and genomic level, where they display significant inter- and intra-tumour heterogeneity [11]. In other words, GBM should not be thought of as a single cancer entity. The Cancer Genome Atlas (TCGA) network outlines four distinct molecular subtypes of GBM: Neural, Proneural, Classical and Mesenchymal [12]. Each of these subtypes harbours unique molecular and genetic aberrations that drive tumorigenesis. Some of these potential tumorigenic processes are discussed below.

## 2. Gliomagenesis

Loss of cell cycle control is involved in gliomagenesis. Cells go through several coordinated processes in their life cycle: cell growth, DNA replication and cell division. Regulation of the cell cycle through checkpoints is the key to normal growth and differentiation. The G1 checkpoint controls the transition from the G1 phase to the S phase, whereas the M checkpoint regulates transition through mitotic phases [13]. The G1 checkpoint has been an important topic of research in GBMs, and it mainly involves cyclins and cyclin-dependent kinases (CDK). Simply, CDKs rely on activation with cyclins to phosphorylate downstream proteins and allow initiation of DNA synthesis. The vast majority of GBMs show alteration in at least one component of such a pathway called p16INK4a/CDK-4/Retinoblastoma 1 (RB1) [14]. In normal cells, cyclin D1 activates CDK-4, which phosphorylates *RB1*, an oncogene, and causes the release of the E2F transcription factor, which initiates G1/S transition [15]. *RB* expression is found to be downregulated in 78% of GBM patients [12]. G1 checkpoint regulation is complex/multi-factorial and acts as a quality control mechanism. During the G1 phase, if DNA damage is detected at the end of the M phase, cyclin-dependent kinase inhibitors may be activated by Tumour protein p53 (p53) to halt the cell cycle and lead to apoptosis to prevent tumorigenesis [13]. Not surprisingly, 87% of GBM patients have an associated *p53* mutation [12]. In summary, cell cycle checkpoints are essential in initiating DNA repair pathways in response to genotoxic stress [16].

In non-neoplastic cells, DNA repair aims to prevent genetic instability that could lead to tumorigenesis. Therefore, it is not surprising that cancer cells often display epigenetic or genetic alterations in the DNA repair pathways [17]. Most notably, p53 mutations leading to impaired apoptotic response have been linked to impaired DNA damage response (DDR) and gliomagenesis [18]. DDR describes the sophisticated cellular mechanisms that detect, signal and repair DNA lesions inflicted by endogenous and exogenous genotoxins. Paradoxically, tumour cells rely on residual DNA repair mechanisms to cope with endogenous stress induced by increased cellular metabolism, mitotic stress and hypoxia, as well as exogenous stress induced by chemotherapy and radiotherapy. Treatment strategies for glioblastoma rely on inducing DNA damage. Ionizing radiation (IR) works mainly by inflicting double-strand breaks (DSBs), base damage and single-strand breaks (SSBs), whereas Temozolomide (TMZ), an alkylating chemotherapy agent, induces N7-methylguanine (N7-meG) and N3-methyladenine [19]. DNA damage caused by these therapies is often repaired by multi-protein pathways such as homologous recombination (HR), non-homologous end joining (NHEJ), or base excision repair (BEJ), with the exception of a repair mechanism via O6- methylguanine-DNA methyltransferase (MGMT) which works as a single protein pathway [20].

Cancer cells adapt DNA repair mechanisms to play a crucial factor in development of therapy resistance and tumour relapse. DNA damage repair deficiency (DDRd) creates predictable and traceable nucleotide alterations in the tumour’s genetic code, creating mutational signatures. Understanding and taking advantage of DDRds could help develop further therapeutic strategies. An established concept of this is identifying MGMT promoter CpG methylation status in treatment of GBM. Forty-five percent of GBM cases are associated with MGMT promoter CpG methylation, which prevents the repair of DNA damage induced by TMZ and thus is associated with increased sensitivity to TMZ and better prognosis [21]. Glioblastoma cells show over-expression of certain growth factors and their receptors. This creates an autocrine growth-promoting loop which provides growth advantage to rapidly proliferating tumour cells. Tyrosine receptor kinases (RTK) such as epidermal growth factor receptor (EGFR) and platelet-derived growth factor (PDGF) have been associated with GBM [22]. Two proliferating signalling cascades have been found to be over-active in glioblastomas: PI3K/Akt/mTOR and Ras/Raf/MAPK Pathways. Interestingly, there is evidence in the literature that there is a mutually inhibitory crosstalk between these two pathways [23].

### 2.1. PI3K/Akt/mTOR Pathway

**Figure 1:**
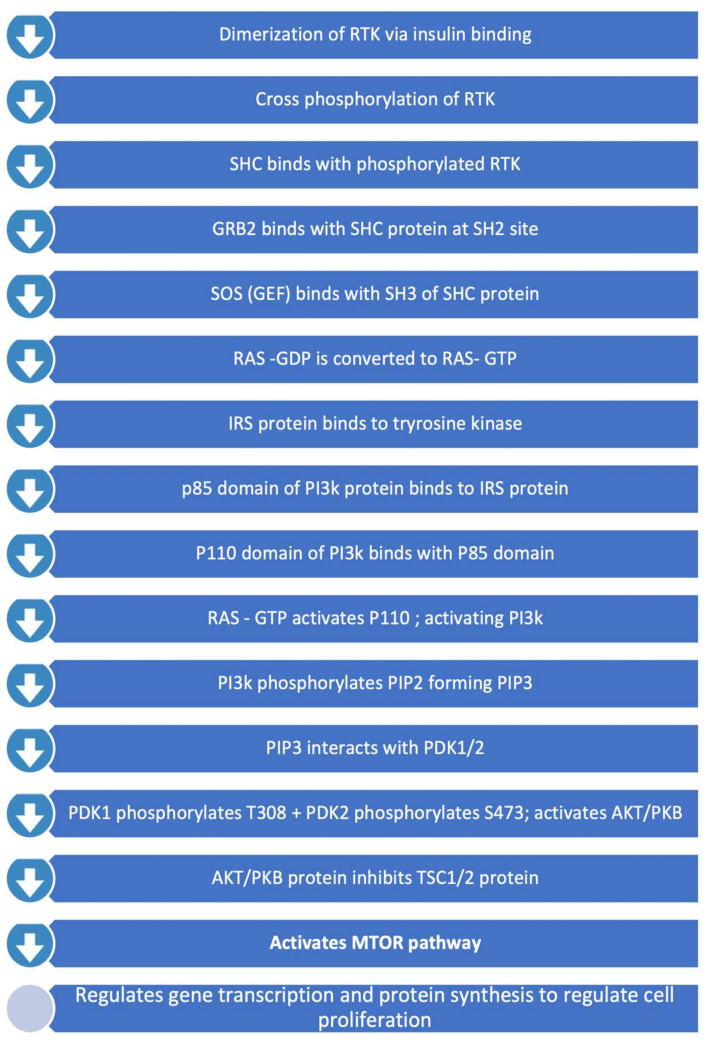
PI3K/Akt/mTOR Pathway.

PIK3/Akt/mTOR Signalling Pathway is involved in various cellular processes including proliferation, growth, cytoskeletal rearrangement and apoptosis [12]. This pathway, summarised in Figure 1, involves RTKs (such as EGFR) and tumour suppressor protein PTEN (Phosphatase and Tensin Homolog Deleted from Chromosome 10). PTEN is a lipid phosphatase and leads to downregulation of the RTK/PI3K/Akt pathway via antagonizing PI3K function [24]. Protein kinase B (Akt) and mammalian target of rapamycin (mTOR) are serine/threonine-specific protein kinases (STKs) that play key roles in cellular proliferation (Figure 1) [10]. RTK/PI3K/Akt signalling is altered in 80% of human GBMs. Homozygous deletion or mutations of *PTEN* leading to constitutive activation of this pathway were associated with 40% of GBMs [12]. Mutations and amplifications in the *PIK3CA,* coding for p110 subunit, have been associated in 5% and 13% of primary GBMs, respectively [25].

### 2.2. RAS/RAF/MAPK Pathway

**Figure 2:**
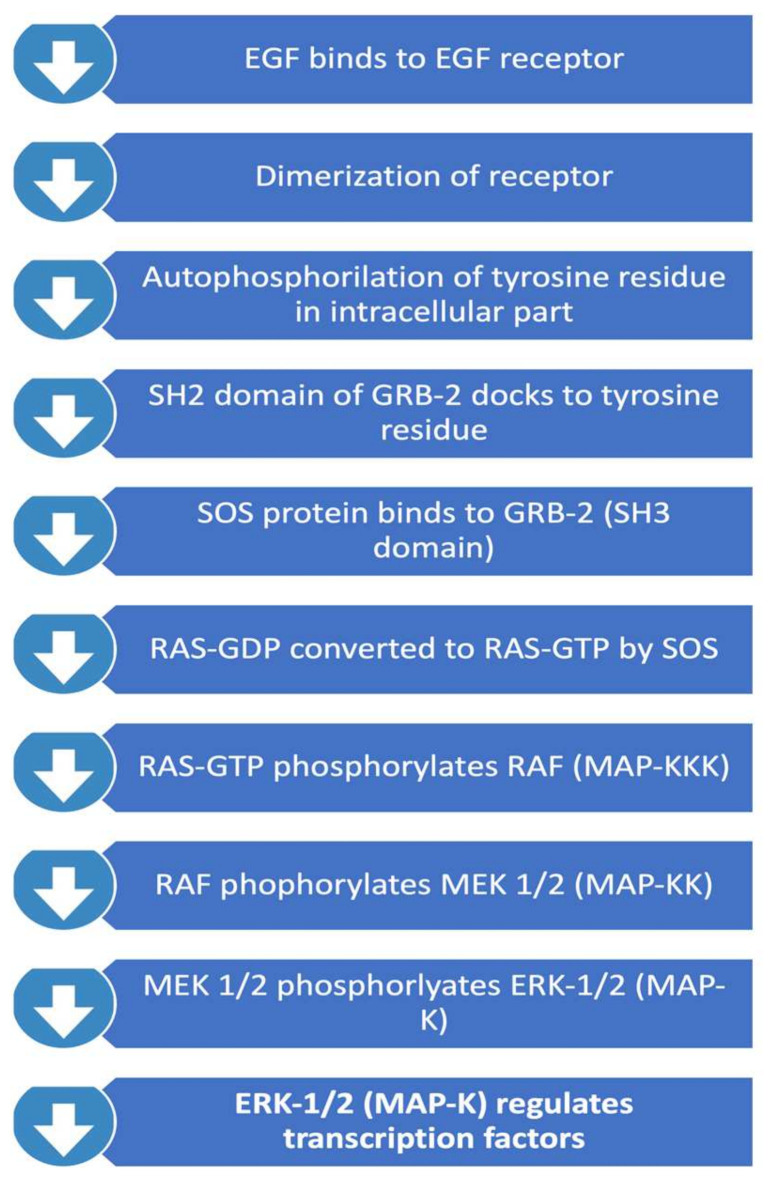
RAS/RAF/MAPK Pathway.

This pathway, summarised in Figure 2, is important in regulating cell proliferation, differentiation, and survival. Activated RAS protein recruits the RAF family of serine/theorinine kinase (STKs) such as ARAF, BRAF and CRAF1. Activated RAF subsequently activates mitogen-activated protein kinase kinase called MEK. Active MEK phosphorylates mitogen-activated protein kinase (MAPK). MAPK subsequently translocates into the nucleus and leads to activation of important transcription factors that promote cell proliferation. Important transcription factors activated through this pathway include c-myc, STAT (signal transducers and activators of transcriptome) and PPARγ (peroxisome proliferator-activated receptor γ) which are involved in inducing cell cycle progression and anti-apoptosis genes [26]. Interestingly, RAS mutations themselves are rare in GBMs, and therefore focus has been on the upstream regulators of the pathway. RAS activation is regulated by neurofibromin 1 (NF-1), a tumour suppressor gene. NF-1 has been identified to play a role in tumorigenesis in gliomas. 18% of GBMs contain NF-1 mutation/homozygous deletion, whereas this number is higher in mesenchymal GBMs (37%) [12].

## 3. Invasion and Migration

GBM remains difficult to treat because these cells employ multiple cellular and metabolic mechanisms to survive and spread. GBM can quickly adapt to adverse conditions in their environment, including those induced by radio or chemotherapy. These cells are also able to evade the immune system and continue to progress and invade. Rapidly growing GBM cells have increased metabolic and nutritional demands. This creates microenvironmental stressors such as hypoxia and metabolic scarcity. In response to these stressors, GBM cells undergo an “angiogenic switch” where new blood vessels are established through upregulation of proangiogenic factors, cytokines and relevant receptors [27]. Angiogenic factors such as vascular endothelial growth factor (VEGF) over-expression has been associated with GBM angiogenesis. Glioblastoma cells demonstrate altered expression of cytoskeletal proteins, signalling molecules and cell surface receptors [28]. Epithelial-to-mesenchymal transition (EMT) is initiated by growth factors [29] and involvement of the PI3K/AKT pathway [30]. Genetic make-up of these mesenchymal migratory cells includes high expression of proteins such as N-cadherin and fibronectin [31] which allow mobility and invasiveness of glioma cells [32].

Some GBM cells continue to adapt and evolve by preserving their stem cell abilities. These glioma stem cells (GSC) express antigens such as Nestin, CD133 (prominin-1), Musashi-1 and Bmi-1 that are specific to neural stem and progenitor cells [33]. GSCs are able to self-renew, remain multi-potent and initiate tumour growth when implanted to animal models. Expression of two main neural progenitor regulators, sonic hedgehog homolog (SHH) and Notch, have been identified to be altered or overexpressed in GSCs [34]. Similarly, aberrant IL6/JAK/STAT3 pathway has been associated in GBM. IL6 regulates Janus kinase (JAK) activation of STAT3 transcription factors (family of signal transducers and activators of transcription), which is required to maintain pluripotency of neural stem cells. STAT3 activation is observed in GBM and is associated with increased tumour grade [35].

Cancer cells use various mechanisms to circumvent host immune surveillance. GBM-associated endothelial cells create an immunological barrier around the tumour microenvironment by modulating immune checkpoints [36]. An immune checkpoint protein called CD200 has been associated with this process and found to be upregulated in GBM. CD200 causes suppression of pro-inflammatory cytokines such as IL2 and IFNy [36].

Another immunosuppressive mechanism potentially employed by GBM is through suppression of dendritic cell (DC) function. DC function plays an integral part in initiating adaptive immune response against cancer cells by antigen uptake and presentation and co-stimulation of T cells [37]. Glioblastoma microenvironments contain factors that can potentially suppress DC function; growth factor-beta (TGF-β) and indoleamine 2,3-dioxygenase (IDO) [38]. With these types of adaptations, GBMs are able to remain out of reach of the immune system and continue to proliferate.

## 4. Materials and Methods

A systematic search of the literature was performed in accordance with PRISMA guidelines for molecular pathogenesis and therapeutic advances. The search was done in the PubMed library and the results were filtered for the last ten years, in English, French or German language. Our study included original and meta-analysis studies that discussed (i) molecular diagnosis and prognosis, (ii) pathogenesis, (iii) gliomagenesis, (iv) infiltration/migration, (v) immune system and (vi) signalling pathways of the GBM. We have excluded duplicate articles, reviews and systematic reviews, studies about paediatric population (<18 years), low grade gliomas (WHO grade I, II) and anaplastic gliomas (WHO grade III) from our study. The search in molecular pathogenesis aimed to group and elucidate the biological mechanisms behind the pathways and the cell events that are correlated to GBMs. The search for therapeutic advances aimed to provide new knowledge that could help in the treatment of the glioblastoma. A four-stage approach was taken for our search, and it was completed with a search in the references of the articles included in our study. Therefore, a Tier-1 search was done under the terms: “(glioblastoma) AND (signaling pathways)”. The Tier-2 search was done under the search terms: (i) (gliomagenesis) AND (cell migration), (ii) (gliomagenesis) AND (cell survival), (iii) (gliomagenesis) AND (DNA repair), (iv) (gliomagenesis) AND (apoptosis), (v) (gliomagenesis) AND (G2/M arrest), (vi) (gliomagenesis) AND (G1/S progression), (vii) (gliomagenesis) AND (cell cycle progression). The Tier-3 and Tier-4 searches were done under the terms: “(glioblastoma) AND (theranostics)”, and “(glioblastoma) AND (vaccine)” respectively. Our results were screened by title and abstract for exclusion criteria. Whole articles that met our inclusion criteria were sought, or there was not enough data to apply exclusion criteria. The reference list of the articles included in our systematic review was further screened for additional studies.

## 5. Results

**Figure 3:**
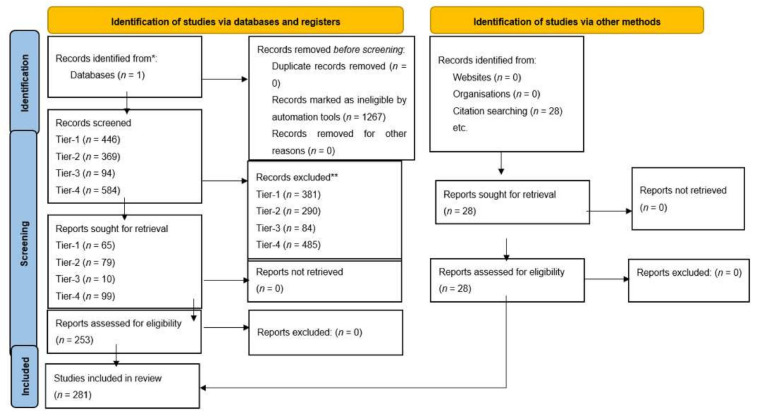
Prisma 2020 Flow-chart [72].

Tables demonstrate search terms used in PubMed, results from initial search with filters and articles meeting inclusion criteria (Figure 3). See Table 1 for selected articles relating to molecular pathogenesis and future treatment perspectives of GBM. All articles included in the study can be found in Tables S1–S9 in the Supplementary Materials. From our search in signalling pathways in GBM, we included eight articles which met the inclusion criteria for PI3K class 1/AKT signalling. These focused on targeting PI3K regulators such as WDR81 [39] to promote autophagy in GBM cells or inhibiting specific isoforms of PI3K to attenuate gliomagenesis. Moreover, blockade of downstream pathways in combination with PI3K had a synergistic effect on reducing tumour growth [40]. Expectedly, metabolic pathways which activate glycolysis were up-regulated secondary to PI3K dependent pathways [41]. Similarly, to PI3K Class 1, we included four articles for PI3K Class 2 which referred to effects of PI3K isoform and regulator inhibition, in addition to altered metabolism associated with this pathway. Four articles discussing the RTK/RAS pathway were analysed, and two studies highlighted the role of CIC as a tumour suppressor protein whereby preventing degradation or inhibition sensitised GBM cells to its anti-tumorigenic effects [42,43]. Four studies relating to the RB pathway were used in this review, two of which examined the use of CDK inhibitors in clinical trials, showing these agents to be ineffective monotherapy due to lack of progression free survival [44,45]. Forty-five articles examining the p53 pathway were included which reviewed the mechanisms to reactivate and enhance tumour suppressor activities of p53. For cell migration, our search yielded 13 articles which met the inclusion criteria and discussed the use of the actin cytoskeleton and MMP to promote cell migration in GBMs [46,47,48]. There is a significant role of RNA–long non-coding, micro or circular to regulate cell motility; downregulation of specific RNAs can suppress migration. EMT is augmented by alterations in the β-Catenin/Wnt pathway and expression of proteins such as E-cadherin and Vimentin [49,50,51]. Fourteen articles pertaining to cell survival were included. Glucose uptake is a key to cellular function, this is up-regulated in GBM cells by many mechanisms, one of which includes overstimulation of α-Ketoglutarate activated by NF-κB signalling, leading to increased uptake [52]. Overexpression of transcription factors such as NF-Y was associated with cell survival [53]. Only four articles related to DNA repair were analysed. DNA damage repair deficiency was most commonly found in p53 [54]. Targeting DNA repair mechanisms such as PARP attenuated gliomagenesis [55] and inhibition of DNA repair protein such as RAD51 in GBM cells resulted in greater TMZ sensitivity [56]. Two articles associated with G2/M cell cycle arrest were included, which discussed blocking zinc finger protein resulting in reduced tumour growth and enhanced apoptosis [57]. For apoptosis, 29 articles were included; the search identified specific RNAs involved in regulation and mediation of apoptosis and detection of therapeutic drugs that potentially reduce tumour growth. Proteins such as heterochromatin, including cell surface protein, have a role in apoptosis. G1/S Progression search yielded two studies, one of which identified an important role of TROAP in cell cycle regulation and enhanced tumour progression [58]. Cell Cycle Progression search yielded 19 articles relevant to cell cycle progression. In our search for cutting edge advances in therapy, the field of theragnostics contributed to our results ten articles. This new technology included specific long non-coding RNAs that could be identified and targeted by existing drugs in GBM patient population subtypes [59]. Moreover, EGFRvIII targeted chimeric antigen receptor T (CAR-T) cell therapy response was also discussed [60]. Finally, 99 articles related to vaccines in GBM were included. Vaccines induced immune response predominantly through dendritic cells and T cells to seize innate antitumorigenic characteristics and prevent tumour progression and permit longer survival.

## 6. Discussion

What cellular and molecular events lead to the development of glioblastoma, and can we intervene in this process at any step? The key to treating GBM and improving patient survival likely lies in answering these questions. That is why, in this systematic review, we highlight the recent advancements in our understanding of the molecular pathogenesis of GBM and discuss related novel treatment strategies.

### 6.1. Gliomagenesis

The most widely accepted hypothesis of GBM development, referred to as gliomagenesis, suggests that GBM develops from mature glia cells undergoing neoplastic transformation through aberrant cellular and molecular events. This theory traditionally focused on the glial cells as a cell of origin because they were thought to be the only dividing cells in the adult brain. However, we now know that the adult brain retains neural stem cells and glial progenitor cells which are also susceptible to neoplastic transformation. In fact, the vast level of heterogeneity observed amongst gliomas may suggest that they develop from a single but multipotent progenitor cell [73]. Neural stem cells exhibit characteristics similar to that of GBM cells; high motility, possession of immature antigenic phenotypes and ability to activate developmental signalling pathways [74]. This active state makes them more prone to malignant transformation requiring fewer mutations compared to differentiated glial cells [75]. Maintaining cancer stem cell (CSC) traits, often referred to as stemness, can promote and sustain tumorigenesis. Supporting this cancer stem cell theory, new evidence suggests that GBM cells often show overexpression of common neural progenitor-cell markers such as nestin, CD133 and CD163 [76,77,78]. These markers may play an essential role in tumour initiation. Singh et al., in 2004, showed that transplanting CD133-expressing GBM cells into mouse brain was able to produce a tumour with preserved parent histological appearances [79]. This, however, was not observed when CD133 negative cells were used [79].

Targeting progenitor-cell marker CD133 presents a novel therapeutic strategy to inhibit GBM stemness and potentially halt gliomagenesis early on. In 2019, Huang et al. showed that the AP-2α transcription factor, which is often downregulated in GBM cells, reduces CD133 expression through Nanog/Sox2/CD133 axis [65]. The study demonstrated that restoring AP-2α expression with the use of miR-26a inhibitors not only suppressed glioma stem cells’ self-renewal abilities in vitro, but also inhibited subcutaneous and intracranial xenograft tumour growth in vivo [65]. This study suggests AP-2α is responsible for maintaining GBM stemness via Nanog/Sox2/CD133 and could serve as a novel therapeutic target. More recently, CD133 has been a target for novel immunotherapy approaches. Do et al. developed a dendritic cell (DC)-based vaccine against CD133 expressing GBM cells by transfecting CD133 mRNA into DC [80]. Immunization in humanised mouse models led to a potent immune response against CD133 positive GBM cells and showed abrogated tumour progression in vitro and in vivo [80]. This suggests CD133 mRNA-transfected DC vaccination could be valuable as an immunotherapy against cancer stem cells.

GBM development could also be inhibited by targeting metabolic pathways involved in gliomagenesis. Relevant signalling pathways such as PI3K/Akt/mTOR and Ras/Raf/MAPK have already been discussed in detail in the background section. Our review showed extensive research on developing therapies against the PI3K signalling pathway. Xu et al. in 2019, studied the anti-proliferative and apoptotic effects of PI3Kβ isoform inhibitor AZD6482 on PTEN-mutated GBM cell lines [81]. AZD6482 administration showed a dose-dependent cytotoxicity to glioma cells leading to increased apoptosis and cell cycle arrest at the G1 phase in vitro [81]. This suggested a possible benefit of an isoform specific approach. Jones et al. studied the effects of isoform-specific PI3K inhibition in GBM cancer stem cells with a hypothesis that this inhibition would promote CSC differentiation and reduce proliferation. Two GBM models studied showed differences in their dominant catalytic PI3K isoform. Inhibiting dominant catalytic PI3K isoforms blocked AKT phosphorylation in both cell lines, but unexpectedly led to increased cancer-stem-cell-associated gene expression. They speculated that this unexpected result could have been caused by inhibitory cross-talk between PI3K and MEK/ERK pathways, where MEK/ERK pathway “corrected” the anti-tumour effects of PI3K inhibition [82]. This highlighted the need for combined targets. Supporting this idea, Iqbal et al. in 2016 demonstrated that combining p110α isoform inhibitor with a PIM kinase inhibitor can successfully reduce GBM stemness and GBM neurosphere growth in cultures [83]. Although it presents a novel therapeutic target, PI3K signalling involvement in GBM is complicated and requires further research.

Reprograming CSCs into more differentiated and less oncogenic phenotypes is a novel therapeutic approach in GBM. Undifferentiated CSCs with an uncontrolled renewal potential play a pivotal role in the development and progression of GBM [84]. The potential role of PI3K in this process has already been discussed above. In a different approach, Lee et al. demonstrated that a protein called Znf179 is involved in reprogramming GBM cells into a more differentiated phenotype by inducing cell arrest in the G_0_/G_1_ phase through p53-p21-p27 cell cycle signalling pathways. Interestingly, Znf179′s mechanism of action did not affect apoptosis or expression levels of neural progenitor markers such as CD133. It worked by reducing tumour burden via up-regulating GBM cell differentiation without depleting the neural progenitor cell population [64]. Further research is needed to better understand Znf179′s therapeutic application in human GBMs.

Inducing apoptosis is another potential approach for GBM treatment. Recently, studies have focused on the restoration of p53 function. One of the mechanisms by which p53 function is altered in GBM is through sequestration by MDM2 oncoprotein. Even in the cells with genetically functional p53, formation of the p53/MDM2 complexes reduces p53 availability, and thus, function. Therefore, multiple treatment approaches focused on disrupting these p53/MDM2 complexes. Costa et al., in 2013, demonstrated that a small MDM2 inhibitor molecule called ISA27 was able to reactivate p53 function to initiate cell cycle arrest and apoptosis in vitro. It was also effective in inhibiting cell proliferation and inducing apoptosis of tumour tissue in mouse models [85]. Another study used small-molecule antagonists of MDM2 named nutlin-3a to inhibit p53/MDM2 interaction. The use of nutlin-3a successfully induced p53-dependent apoptosis in glioma cell lines [86]. Disrupting the p53/MDM2 complex using RNA molecules has also been studied. Lou et al., in 2020, identified a circular RNA molecule called CDR1as which, by binding to p53, disrupted p53/MDM2 complex formation. Consequently, CDR1as was effective at inhibiting tumour growth both in vitro and in vivo [87]. It is important to understand that therapeutic agents focusing on p53/MDM2 interaction were only effective in treating those with functional p53. Glioma cells with absent or mutated p53 were insensitive to treatment with these agents [85,86,87].

### 6.2. Invasion and Migration

Cancer cells evolve to survive under stress conditions such as hypoxia and starvation. Targeting these adaptive mechanisms, which are also involved in developing therapy resistance, could be a novel treatment strategy. Wanka et al. studied the effects of a metabolism-regulator called TIGAR (Tp53-induced glycolysis and apoptosis regulator) on hypoxia-induced cell death. TIGAR was overexpressed in GBM and protected the cells against environmental glucose and oxygen restrictions. TIGAR worked by increasing respiration and improving energy yield from glucose. Reversal of TIGAR’s effects was possible with silencing RNA (siRNA)-led inhibition of TKTL1. This study suggests that targeting TKTL1 may reduce tumour survival and increase sensitivity to hypoxia-induced therapies [88]. On the other hand, Wang et al. in 2019 identified that GBM cells could up-regulate GLUT1 via α-KG/IKKβ/NF-κB signalling to cope with low glucose conditions [52]. Under low glucose conditions, phosphorylated GDH1 interacts with the IKK complex to produce α-KG, which directly activates IKKβ and NF-κB signalling and leads to GLUT1 expression. This promotes compensatory glucose uptake [52]. This novel pathway suggests that preventing phosphorylation of GDH1 or disrupting α-KG and NF-κB interaction could be targeted for future therapies. Disrupting GBM adaptations to hypoxia and starvation would decrease its survival and progression.

GBM cells show diffuse infiltration into the surrounding neural network as the disease progresses. This process is complex and include multiple signalling pathways. Here, we will focus on Wnt/β-catenin signalling pathway as a novel therapeutic target. This pathway, which is often aberrantly activated in GBM, promotes epithelial-to-mesenchymal transition (EMT) and tumour invasion through increased β-catenin phosphorylation and/or nuclear localization [89]. EMT is mediated via increased expression of downstream factors such as Twist1, ZEB1, Snail and Slug [90]. Bao et al. studied the effects of Wnt/β-catenin inhibition on GBM invasion and migration. They demonstrated that inhibition of the Wnt/β-catenin pathway with CBX7 overexpression led to reduced GBM invasive ability and EMT. This effect was facilitated through CBX7-mediated enhancement of Wnt/β-catenin inhibitor (DKK1) expression, which led to reduced downstream ZEB1 expression in vivo and in vitro [91]. This study offers a potential role of CBX7 in the inhibition of neoplastic development. On the other hand, Liu et al. tested a novel micro-RNA therapy to regulate the Wnt/β-catenin pathway in GBM. Normally, micro-RNA (miR-504) inhibits the Wnt/β-catenin pathway by directly repressing FZD7 expression. Low miR-504 expression and low miR-504/FZD7 ratio correlated with increased MET and poor survival. They demonstrated that increasing miR-504 expression could suppress GBM migration, invasion, EMT and stemness characteristics in vitro and in vivo [92]. This identifies miR-504 as a possible therapeutic candidate in malignant brain tumours.

Glioblastoma cells masterfully manipulate tumour microenvironment (TME) and the cells within to invade brain parenchyma. Astrocytic gliosis is a term used to describe reactive changes seen in previously healthy astrocytes following exposure to glioma environment. These astrocytes show increased proliferation and display properties that help GBM invasion and angiogenesis [93]. Recently, the focus has been on studying the role of GBM extracellular vesicles (EV) in pathological glioma/astrocyte interaction. Hallal et al. exposed healthy astrocytes to EVs isolated from GBM patients [94]. Internalization of GBM-EV by astrocytes led to enhanced astrocyte podosome formation, suggesting a mechanism by which astrocytes support GBM invasion (ECM remodelling and blood-brain barrier breakdown). These effects were more significant when EVs were isolated from cancer stem cells (NES+/CD133+) compared to differentiated GBM cells (NES-/CD133-). Further molecular analysis suggested GBM-EV exposure activated MYC and inhibited p53 pathways, creating a pro-inflammatory and tumour-promoting astrocyte phenotype [94]. A novel immunotherapy approach involving GBM extravesicles has been tested by Pineda et al. Vaccination with GBM microvesicles led to significant reduction in tumour volume in animal models. This was also associated with increased tumour infiltration with immune cells [69].

### 6.3. Immune Suppression

GBM cells can evade the host immune system by modifying TME. Notch1 has been identified as a potential regulator in the development of an immune suppressive TME. Notch1 increases expression of M-CSF and SDF-1, which are important in recruitment and development of TAMs (tumour-associated macrophages). TAMs secrete pro-tumorigenic factors such as TGF-β and promote tumour growth. Gu et al. demonstrated that inhibiting an upstream regulator of Notch1 called NKAP (NF-κB activating protein) led to abrogated tumour growth and invasion both in vitro and in vivo. NKAP’s function involved regulating TME through Notch1 expression [49]. Interestingly, Hai et al. demonstrated that Notch1 and NF-κB interact in a reciprocal regulatory loop. Inhibition of Notch1 using a chemical Notch1 inhibitor (DAPT) was associated with NF-κB (p65) suppression and subsequent inhibition of GBM cell proliferation and increased apoptosis both in vitro and in vivo [95]. This study suggests that, in the future, combining drugs to inhibit both Notch1 and NF-κB pathways may be advantageous.

Multiple immunotherapy strategies have been trialed to train the host immune system against GBM cells. Our review identified that the major focus has been on development of dendritic cell vaccines. Simply, these vaccines are developed by training autologous DC to recognize tumour-specific factors. This is accomplished by pulsing them with tumour lysate or tumour-specific antigens.

One group designed an autologous DC vaccine called ICT-107 using six synthetic peptide epitopes targeting glioma-stem-cell-associated antigens. A randomized, double-blind placebo-controlled phase II trial using ICT-107 showed that the addition of ICT-107 to Stupp Protocol significantly improved progression-free survival with maintenance of quality of life [96]. They further identified that patients with HLA-A2+ tumours showed increased benefit and greater immunological response [96].

Other trials used tumour lysate in the development of autologous DC vaccines [66,97]. Phase I data for such vaccines establishes their safety and tolerability [66]. The phase II trial of such a vaccine (Audencel) showed that although well tolerated, it failed to show significant improvement in overall- or progression-free survival [97]. Blinded interim data for a phase III clinical trial using a similar DC vaccine (DCVax^®^-L) suggests the addition of DCVax^®^-L to standard therapy is safe and may extend survival [98].

Certain molecular characteristics may influence response to DC immunotherapy. Erhant et al. studied effects of Audencel immunization at molecular and cellular levels after it failed to show positive clinical outcomes. They identified that Audencel was able to stimulate the immune system, especially Th-1 related immunovariables, however its effect was influenced by the host immune system [99]. Supporting this theory, Zeng et al. demonstrated that high 90K antigen expression, a biomarker for glioma malignancy and prognosis, was associated with enhanced response to DC vaccine immunotherapy in vitro [100]. A double-blind, placebo-controlled phase II clinical trial showed that patients with wild-type IDH1, mutated TERT and low B7-H4 expression have greater response to DC-based vaccine immunotherapy [101]. Other predictors of beneficial response to autologous DC vaccine were younger age, gross resection of tumour, tumour showing infiltrating lymphocytes and peripheral blood mononuclear cells with lower PD-1+/CD8+ ratio [102].

An alternative immunotherapeutic approach is the use of B cell-based vaccines. Lee-Chang et al. developed BVax, a B cell vaccine made up of 4-1BBL+ B cells activated with CD40 agonist and FNγ stimulation [103]. BVax has been shown to migrate to secondary lymphoid organs and activate autologous CD8+ T cells, which could successfully kill autologous GBM cells. A combination of radiation, BVax and PD-L1 blockade led to 80% tumour eradication and elicited immunological memory. Excitingly, this immunological memory prevented the re-growth of new tumours upon reinjection in cured mice [103]. B cell-based vaccination in GBM treatment is a novel concept and holds great potential for the future. More experimental immunotherapy strategies include the use of viral vectors. Kim et al. developed a novel adenovirus vector carrying a GBM-specific antigen that selectively infected DC. Vaccination with this viral vector in GBM-implanted murine models was associated with prolonged survival [104]. The safety and clinical benefit of these novel therapies are still being investigated.

It is obvious that neuroscience is progressing and shows true potential in finding new drugs against glioblastomas in the future. Neuro-Researchers must deal with the molecular and cellular heterogeneity, the migration and invasion, and the immune microenvironment of the glioblastoma. Another challenge that needs to be taken into consideration is the delivery system of any therapeutic agent to the brain. The significant obstacle to overcome is the blood-brain barrier. The latter is disrupted in glioblastomas, but it is not sufficient to allow all the therapeutic agents to cross the barrier freely [105,106]. Several delivery systems have been described but the research for the optimal one is still young [107]. 

## 7. Conclusions

Recent insights into molecular pathophysiology of GBM has expanded the horizon for therapeutic strategies. In this review, we discussed novel therapeutic strategies targeting every stage of gliomagenesis and GBM progression. Inhibiting glioma cell stemness through the Nanog/Sox2/CD133 pathway can halt gliomagenesis. Potential therapeutic targets for this pathway included miR-26a inhibitors or AP-2α. Proliferation of undifferentiated GBM cells can be stopped by restoration of p53 function through disruption of p53/MDM2 complexes. Utilizing MDM2 inhibitor molecules such as ISA27 and nutlin-3a or p53-binding circular RNA molecule (CDR1as) can restore p53 function and induce apoptosis. Targeting GBM adaptation mechanisms can prevent GBM survival. GBM cells’ adaptation to hypoxia and starvation can be disrupted by targeting T53-induced glycolysis and apoptosis regulator action. Glioma invasion and migration can be targeted through multiple pathways. Glioblastoma cells masterfully manipulate tumour microenvironment to recruit astrocytes into their invasive process. Understanding the role of GBM extracellular vesicles in GBM/astrocyte interaction can help develop novel treatment strategies, including the use of vaccine immunotherapy. Exciting novel immune therapy strategies including dendritic cell vaccines, B-cell vaccines and viral vectors have been developed for the treatment of GBM. Autologous dendritic cell vaccines are already being tested in phase III clinical trials, while others, like viral vectors, are still early in their development stages. Whatever stage they may be in, these novel therapeutic approaches open a door of possibility and hope for the future of GBM treatment.

## Figures and Tables

**Table 1:** Selected articles for molecular pathogenesis and future perspectives for glioblastoma.

Article Title	Authors	Journal	PMID	Year	Outcome
The Circ_0001367/miR-545-3p/LUZP1 Axis Regulates Cell Proliferation, Migration and Invasion in Glioma Cells	Dong et al. [46]	Frontiers in Oncology	34869035	2021	Down-regulation of circ_0001367 reduces invasion, migration and proliferation.
IDH1 R132H Mutation Enhances Cell Migration by Activating AKT-mTOR Signaling Pathway but Sensitizes Cells to 5-FU Treatment as NADPH and GSH Are Reduced.	Zhu et al. [51]	PLoS	28052098	2017	IDH1 R132H mutation promotes AKT-mTOR pathway to increase MMP and β-Catenin reduce E-cadherin.
Overexpression of SCLIP promotes growth and motility in glioblastoma cells	Zhang et al. [61]	Cancer Biology Therapy	25511414	2015	SCLIP (microtubule-destabilizing protein) is detected at high levels in GBMs augments migration. STAT3 is required for maintaining SCLIP stability.
Whole exome sequencing-based analysis to identify DNA damage repair deficiency as a major contributor to gliomagenesis in adult diffuse gliomas	Ege Ülgen et al. [54]	Journal of neurosurgery	30952131	2019	DNA damage repair deficiency (DDRd) are predictable and traceable nucleotide alterations in GBMs. Suggests DDRd may be important for gliomagenesis.
Propofol inhibits Wnt signaling and exerts anticancer activity in glioma cells	W. Xu et al. [62]	Oncology Letters	29928428	2018	The results indicate a novel mechanism of anticancer activity for propofol and provide novel insights into the signaling pathways regulated by propofol. Potential use of propofol as a novel agent for the treatment of patients with glioma.
The Inhibition of microRNA-128 on IGF-1-Activating mTOR Signaling Involves in Temozolomide-Induced Glioma Cell Apoptotic Death	Peng-Hsu Chen et al. [63]	PLOS one	27893811	2016	miR-128-inhibited mTOR signaling is involved in TMZ-mediated cytotoxicity. Better understanding of cytotoxic mechanisms of TMZ involved in glioblastoma development.
TROAP regulates cell cycle and promotes tumor progression through Wnt/β-Catenin signaling pathway in glioma cells	Zong-Qing Zhao, et al. [58]	CNS Neuroscience & Therapeutics	34077623	2021	TROAP ((Trophinin Associated Protein) accelerated the progression of gliomagenesis through Wnt/β-Catenin pathway, and TROAP might be considered as a novel target for glioma therapy.
Znf179 induces differentiation and growth arrest of human primary glioblastoma multiforme in a p53-dependent cell cycle pathway	Kuen-Haur Lee et al. [64]	Nature	28684796	2017	Znf179 induces neuronal differentiation. p53-p21-p27 cell cycle signaling pathways are involved in Znf179-induced differentiation of GBM cells. Glioma patients with higher Znf179 expression levels have longer survival rates
The miR-26a/AP-2α/Nanog signaling axis mediates stem cell self-renewal and temozolomide resistance in glioma	Wenhuan Huang et al. [65]	Theranostics	31534499	2019	AP-2α reduces the stemness and TMZ resistance of glioma by inhibiting the Nanog/Sox2/CD133 axis and IL6/STAT3 signaling pathways.
A Phase I Study of Autologous Dendritic Cell Vaccine Pulsed with Allogeneic Stem-like Cell Line Lysate in Patients with Newly Diagnosed or Recurrent Glioblastoma	Hu et al. [66]	Clinical Cancer Research	34862245	2021	Autologous dendritic cell vaccines pulsed with lysate originating from GBM stem-like cell lines was safe and without significant side effects.
Identification of Tumor Antigens and Immune Landscape in Glioblastoma for mRNA Vaccine Development	Ye at al. [67]	Frontiers Genetics	34527020	2021	Prognostic mRNA antigens: ARPC1B and HK3 identified from TCGA were positively correlated with antigen presenting cells in GBMs validating role in immune response. Immune subtype 2 had “pro-inflammatory features” better response to vaccines.
Malignant Glioma Therapy by Vaccination with Irradiated C6 Cell-Derived Microvesicles Promotes an Antitumoral Immune Response	Benjamiín Pineda et al. [68]	Molecular Therapy	31204210	2019	Vaccination with irradiated cell-derived microvesicles led to reduction of tumour volume and increased immune cell infiltration of the implanted tumour tissue.
Actively personalized vaccination trial for newly diagnosed glioblastoma	Hilf N et al. [69]	Nature.	30568303	2019	Phase I trial GAPVAC-101 of the Glioma Actively Personalized Vaccine Consortium (GAPVAC). Promising results i.e., responses of central memory CD8+ T cells and CD4+ T cell responses of T helper 1 type against predicted neoepitopes.
Rindopepimut with temozolomide for patients with newly diagnosed, EGFRvIII-expressing glioblastoma (ACT IV): a randomised, double-blind, international phase 3 trial.	Weller M et al. [70]	Lancet Oncol.	28844499	2017	Rindopepimut is a vaccine targeting the EGFR deletion mutation EGFRvIII. Phase 3 trial to explore if the addition of rindopepimut to chemotherapy did not increase survival in newly diagnosed GBM.
Immunomodulation Mediated by Anti-angiogenic Therapy Improves CD8 T Cell Immunity Against Experimental Glioma	Malo CS et al. [71]	Frontiers in Oncology	30211113	2018	VEGF-Trap treatment has anti-angiogenic effect, normalizing the vasculature and enhance tumor antigen-specific CD8T cell response.

## Data Availability

Not applicable.

## Supplementary Materials

The following are available online at www.mdpi.com/article/10.3390/ijms23052607/s1.

## Abbreviations

Akt	Protein kinase B
ALDOA	Fructose-bisphosphate aldolase A
ARST	ALDOA-Related Specific Transcript
ASK1	Apoptosis signal-regulating kinase 1
ATIP1	AT2 receptor interacting protein
BCNU	carmustine
BEJ	base excision repair
CAR-T	chimeric antigen receptor T
CBX 7	Chromobox 7
CCND2	Cyclin D2
CCNU	lomustine
CDK	Cyclin dependent kinase
CDR1	Cerebellar degeneration-related protein
CIC	Capicua (Transcriptional suppressor)
CK2	Casein kinase-2
CRISPR	clustered regularly interspaced short palindromic repeats
CRNDE	Colorectal Neoplasia Differentially Expressed
CSC	cancer stem cell
CXCR4	Chemokine receptor-4
DAMPs	Damage-associated molecular pattern
DC	Dendritic cells
DDR	DNA damage response
DDRd	DNA damage repair deficiency
DSBs	double-strand breaks base
Ebp1	ErbB3-binding protein 1
EGFR	Epidermal growth factor receptor
EMT	Epithelial-to-mesenchymal transition
EV	Extracellular vesicles
Fbxw7	F-box/WD repeat-containing protein 7
GAP	GTPase-activating proteins
GDH1	Glutamate dehydrogenase 1
GLUT 1	Glucose transporter 1
GSC	glioma stem cells
GSK-3β	Glycogen synthase kinase-3β
HMGA	High mobility group A
HR	homologous recombination
IDO	indoleamine 2:3-dioxygenase
IKK	IκB kinase
IR	Ionizing radiation
JNK	c-Jun N-terminal kinases
MAPK	mitogen-activated protein kinase
MCL-1	Myeloid leukemia 1
MDM2	Mouse double minute 2 homolog
MDM4	Mouse double minute 4 homolog
MEK	Mitogen-activated protein/extracellular signal-regulated kinase
MGMT	O6- methylguanine-DNA methyltransferase
MHY-9	Myosin Heavy Chain 9
MiR	micro RNA
MK 2	MAPK-activated protein kinase 2
MMP	Matrix Metalloproteinase
mTOR	mammalian target of rapamycin
MTSG1	Mitochondrial tumour suppressor
MTUS1	TUS1 (Microtubule Associated Scaffold Protein 1)
NDRG1	N-myc downstream-regulated gene 1
NF-1	neurofibromin 1
NHEJ	non-homologous end joining
NHEJ	Non-homologous end joining
NKAP	NF-κB activating protein
p53	Tumour protein p53
PARP	Poly (ADP-ribose) polymerase
PDGF	platelet-derived growth factor
PDK1	phosphoinositide-dependent kinase-1
PFKP	Platelet isoform of phosphofructokinase
PI3K	Phosphatidylinositol 3-kinase
PIM	Proto-oncogene serine/threonine-protein kinase
POU	Pituitary specific Pit1, Octamer transcription factor protein, Unc-86 transcription factor
PPARγ	peroxisome proliferator-activated receptor γ
PPDR	Pleiotropic Drug Resistance
Pt-1-DMCa	Platinum analogue Pt-MBL-III_7-D_MCa
PTEN	Phosphatase and Tensin Homolog Deleted from Chromosome 10
RAD 51	DNA repair protein RAD51
RB1	p16INK4a/CDK-4/Retinoblastoma 1
RNAi	RNA interference
RTK	Receptor Tyrosine kinase
RTK	Tyrosine receptor kinases
Sh RNA	Short hairpin RNA
SHH	sonic hedgehog homolog
siRNA	Small interfering RNA
SIRT1	Sirtuin 1
SNRPG	Small Nuclear Ribonucleoprotein Polypeptide G
Sox	SRY-Box Transcription Factor
SSBs	Single-strand breaks
STAT	Signal transducers and activators of transcriptome
STKs	specific protein kinases
TAMs	tumour-associated macrophages
TCGA	The Cancer Genome Atlas
TGF-β	growth factor-beta
THTMP	2-[(3:4-dihydroquinolin-1(2H)-yl)(p-tolyl)methyl]phenol
TIGAR	Tp53-induced glycolysis and apoptosis regulator
TKTL1	Transketolase Like 1
TME	tumour microenvironment
TMZ	Temozolomide
TNFα	Tumour Necrosis Factor α
TSG	Tumour suppressor genes
VEGF	vascular endothelial growth factor
