# Supplementary material for: Molecular Pathogenesis of Glioblastoma in Adults and Future Perspectives: A Systematic Review
Source: Int J Mol Sci. 2022 Feb 26;23(5):2607. doi: 10.3390/ijms23052607 (PMC8910150; doi:10.3390/ijms23052607)
Supplement: Supplementary file 1 [file ijms-23-02607-s001.zip › Full Search Results Tables.pdf]

Table S1. Tier-1 search results for signaling pathways.

Article Title	Authors	Journal	PMID	Year	Outcome
HMGA1 stimulates MYH9-dependent ubiquitination of GSK-3β via PI3K/Akt/c-Jun signaling to promote malignant progression and chemoresistance in gliomas.	Que et al.	Cell death and disease	34887392	2021	HMGA promotes MYH-9 expression which then potentiates tumourgenic effect including migration through GSK-3β with stimulation likely through β-catenin.
The Circ_0001367/miR-545-3p/LUZP1 Axis Regulates Cell Proliferation, Migration and Invasion in Glioma Cells	Dong et al.	Frontiers in Oncology	34869035	2021	Down-regulation of circ_0001367 (circular RNA) and LUZP1 (regulation of actin cytoskeleton) was demonstrated in GBM cells; both act to reduce invasion, migration and proliferation.
SPAG5 Is Involved in Human Gliomagenesis Through the Regulation of Cell Proliferation and Apoptosis	Wang et al.	Frontiers in Oncology	34796102	2021	Sperm-associated antigen 5 (SPAG5) oncogene knockdown reduced proliferation and colony formation, additionally it inhibits migration and invasion.
A novel lncRNA ARST represses glioma progression by inhibiting ALDOA-mediated actin cytoskeleton integrity	Sun et al.	Journal of experimental and clinical cancer research	34099027	2021	ARST operates as a tumour suppressor by disrupting interaction between ALDOA and actin cytoskeleton leading to rapid cofilin-dependent loss of F-actin stress fibers inhibiting cell migration and invasion.<br><br>Fructose-bisphosphate aldolase A (ALDOA)<br>ALDOA Related Specific Transcript (ARST)
Decreased expression of miR-410-3p correlates with poor prognosis and tumorigenesis in human glioma	Wang et al.	Cancer Management and Research	31908530	2019	Blockage of miR-410-3p significantly facilitated migration, enhanced expression of MMP9 and vimentin

NKAP alters tumor immune microenvironment and promotes glioma growth via Notch1 signaling	Gu et al.	Journal of Experimental and Clinical Cancer Research	31277684	2019	NKAP knockdown caused less cell migration and inhibited EMT by drastically reducing N-cadherin, Twist1 and Vimentin.
Zeb1 potentiates genome-wide gene transcription with Lef1 to promote glioblastoma cell invasion	Rosmaninho et al.	EMBO Journal	29903919	2018	Inhibition of Zeb1 (EMT inducing factor) reduced GBM cell migration mediated by Prex 1 (guanine nucleotide exchange factor).
IDH1 R132H Mutation Enhances Cell Migration by Activating AKT-mTOR Signaling Pathway, but Sensitizes Cells to 5-FU Treatment as NADPH and GSH Are Reduced.	Zhu et al.	PLoS	28052098	2017	IDH1 R132H mutation promotes AKT-mTOR pathway to increase MMP and β-Catenin reduce E-cadherin.
CBX7 negatively regulates migration and invasion in glioma via Wnt/β-catenin pathway inactivation	Bao et al.	Oncotarget	28388562	2017	CBX7 (Polycomb-group proteins) overexpression prevents EMT and migration through interaction with Wnt/β-catenin pathway. It reduces expression of MMP and cadherins.
RasGRP3 regulates the migration of glioma cells via interaction with Arp3	Lee et al.	Oncotarget	25682201	2015	RasGRP3 (Ras protein) activity is upregulated in GBM, it interacts with Arp3 (actin-related protein), to promote actin polymerisation and cell migration.
Overexpression of SCLIP promotes growth and motility in glioblastoma cells	Zhang et al	Cancer Biology Therapy	25511414	2015	SCLIP (microtubule-destabilizing protein) is detected at high levels in GBMs augments migration. STAT3 is required for maintaining SCLIP stability.
NOTCH3 is a prognostic factor that promotes glioma cell proliferation, migration and invasion via	Alqudah et al.	PLoS One.	24143218	2013	NOTCH overexpression in GBM increased cell migration.

activation of CCND1 and EGFR					
Role of histone lysine methyltransferases SUV39H1 and SETDB1 in gliomagenesis: modulation of cell proliferation, migration, and colony formation	Soyropoulou et al.	Neuromolecular Medicine	23943221	2013	Inhibiting H3K9 - Histone methyltransferase leads to reduced GBM cell migration

Table S2. Tier-2 search results for cell survival.

Article Title	Authors	Journal	PMID	Year	Outcome
Investigation of simian virus 40 (SV40) and human JC, BK, MC, KI, and WU polyomaviruses in glioma.	Limam S, Missaoui N, Bdioui A, et al.	J Neurovirol.	32124265	2020	Although some polyomaviruses (SV40, JCPyV and MCPyV) were found in GBM sample it did not show they were implicated in gliomagenesis. Detection of these as passenger viruses may allude to their ability to replicate more easily in GBM cells.
Chondroitin sulfate content and decorin expression in glioblastoma are associated with proliferative activity of glioma cells and disease prognosis.	Tsidulko AY, Kazanskaya GM, Volkov AM, et al.	Cell Tissue Res.	31773303	2020	Chondroitin sulphate (CS) proteoglycans such as decorin was overexpressed in GBM cells, raised content was associated with high cell proliferation. Decorin was negatively correlated with patient survival rates. However, high overall CS content was linked with better survival suggesting variability within CS subtypes.
α-Ketoglutarate-Activated NF-κB Signaling Promotes Compensatory Glucose Uptake and Brain Tumor Development.	Wang X, Liu R, Qu X, et al.	Mol Cell.	31447391	2019	Glucose is upregulated by GDH-1 phosphorylation at S384 subunit, interaction with IKK occurs due to low glucose level, this in turn produces alpha ketoglutarate which subsequently activates NK-kB signaling. NF-kB signaling then enhances glucose uptake by the tumor. Under dampened glucose levels IDH-1 depletion reduces GBM cell viability.
Nuclear Smad6 promotes gliomagenesis by negatively regulating PIAS3-mediated STAT3 inhibition.	Jiao J, Zhang R, Li Z, et al.	Nat Commun.	29950561	2018	Cell lines including nuclear Smad6 had quicker cell growth compared with other cell lines. STAT3 was upregulated secondary to Smad6 negative PIAS3 regulation promoting gliomagenesis.

Germline polymorphisms in myeloid-associated genes are not associated with survival in glioma patients.	Jacobs DI, Liu Y, Gabrusiewicz K, et al.	J Neurooncol.	28965162	2018	GBM microenvironment includes myeloid cell lineage which change phenotype to support tumour progression. Single nucleotide polymorphisms (SNPs) were tagged in 91 myeloid associated genes in GBM patients. No correlation was found in between SNPs and survival.
High-dose Neural Stem Cell Radiation May Not Improve Survival in Glioblastoma.	Achari R, Arunsingh M, Badgami RK, et al.	Clin Oncol (R Coll Radiol).	28188088	2017	High dose radiation to Neural Stem cell (NSC), which is usually avoided to maintain plasticity and neurocognition, did not improve survival. Note patients continued standard therapy. Ipsilateral irradiation actually reduced survival rates.
The transcription factor PPARα is overexpressed and is associated with a favourable prognosis in IDH-wildtype primary glioblastoma.	Haynes HR, White P, Hares KM, et al.	Histopathology.	27926792	2017	PPARα is overexpressed in GBM, it did not correlate with prognosis, however it can act as a positive prognostic indicator as examined via TCG.
NF-YC in glioma cell proliferation and tumor growth and its role as an independent predictor of patient survival.	Cui H, Zhang M, Wang Y, Wang Y.	Neurosci Lett.	27495011	2016	Nuclear factor-Y (NF-Y) was positively correlated with GBM grade. Knockdown of NF-YC reduced brain tumor size in vivo.High NF-YC expression was linked with reduced patient survival.
Nuclear unphosphorylated STAT3 correlates with a worse prognosis in human glioblastoma.	Rodrigues BR, Queiroz-Hazarbassanov N, Lopes MH, et al.	Pathol Res Pract.	27013058	2016	Higher nuclear localisation of STAT3 correlated with lower rates of recurrence free and overall survival.
IDH mutation is associated with higher risk of malignant transformation in low-grade glioma.	Leu S, von Felten S, Frank S, Boulay JL, Mariani L.	J Neurooncol.	26780338	2016	IDH mutation has been previously linked to longer patient survival and is one of the earliest events in gliomagenesis. Although its presence improves patient survival rates there is a higher chance of malignant transformation.

Expressions of glia maturation factor-β by tumor cells and endothelia correlate with neovascularization and poor prognosis in human glioma.	Kuang XY, Jiang XF, Chen C, et al.	Oncotarget.	26515590	2016	High levels of Glia maturation factor-β (GMF-β) were associated with higher tumour grade and poor patient prognosis. It promotes gliomagenesis by enhancing neovascularisation.
Pharmacologic Wnt Inhibition Reduces Proliferation, Survival, and Clonogenicity of Glioblastoma Cells.	Kahlert UD, Suwala AK, Koch K, et al.	J Neuropathol Exp Neurol.	26222502	2015	Inhibition of the WNT pathway via porcupine inhibitor LGK974 with elevated AXIN2 which showed WNT activity reduced by 50%. This caused growth suppression, suppressed proliferation and some cell death.
Overexpressed homeobox B9 regulates oncogenic activities by transforming growth factor-β1 in gliomas.	Fang L, Xu Y, Zou L.	Biochem Biophys Res Commun.	24582746	2014	Homeobox B9 is a protein encoding gene on chromosome 17 that regulates gene expression and cell survival. Higher concentration found in GBM. Overexpressed HOXB9 stimulated progression and migration of glioma cells, as well as the tumorigenicity and this was reversed when HOXB9 was knocked down.
Tumorigenesis and prognostic role of hepatoma-derived growth factor in human gliomas.	Hsu SS, Chen CH, Liu GS, et al.	J Neurooncol.	22037800	2012	Knockdown of nuclear Hepatoma-derived growth factor (HDGF) in Glioma cells led to inhibition of cell growth and invasion. HDFG presence is a marker for shorter survival in patients after surgery. Therefore, it can be a prognostic marker and therapeutic target.

Table S3. Tier-2 search results for DNA repair.

Article Title	Authors	Journal	PMID	Year	Outcome
The Tumor Suppressor MTUS1/ATIP1 Modulates Tumor Promotion in Glioma: Association with Epigenetics and DNA Repair	Nikhil Ranjan et al.	Cancers	33809019	2021	*MTUS1*/ATIP1 is downregulated in high grade GBMs and regulates gliomagenesis, progression and therapy resistance. ATIP1 overexpression reduced glioma progression and prolonged survival however, ATIP1 overexpression resulted in radio-resistance.
PARP targeting counteracts gliomagenesis through induction of mitotic catastrophe and aggravation of de-	Jara Majuelos-Melguizo et al.	Oncotarget.	25576921	2015	In PTEN-deficient GBM cells PARP inhibition led to a mitotic catastrophe via inducing severe genomic instability, exacerbated homologous recombination repair (HR) deficiency and down-regulating spindle assembly checkpoint factor BURB1. Combining with EGFR blocker erlotinib retarded gliomagenesis.

ficiency in homologous recombination in PTEN-mutant glioma					
Mutant IDH1-driven cellular transformation increases RAD51-mediated homologous recombination and temozolomide resistance	Shigeo Ohba et al.	Cancer research.	25035396	2014	Mutant IDH-1 is associated with enhanced RAD51-mediated homologous recombination DNA repair (HR) and facilitate Temozolomide (TMZ) resistance. This suggests inhibitors of HR could potentially enhance TMZ response in IDH-1 mutant GBMs.
Whole exome sequencing-based analysis to identify DNA damage repair deficiency as a major contributor to gliomagenesis in adult diffuse gliomas	Ege Ülgen et al.	Journal of neurosurgery.	30952131	2019	DNA damage repair deficiency (DDRd) are predictable and traceable nucleotide alterations in GBMs. Eighty-four percent of tumours had at list 1 DDRd signature, TP53 being the most common somatically altered DDR gene. This suggests DDRd may be important for gliomagenesis.

Table S4. Tier-2 search results for G2/M Arrest.

Article Title	Authors	Journal	PMID	Year	Outcome
Silencing of ZFP36L2 increases sensitivity to temozolomide through G2/M cell cycle arrest and BAX mediated apoptosis in GBM cells	Che Mat et al.	Molecular Biology Reports	33590411	2021	Blocking zinc finger protein 36–like 2 (ZFP36L2) via RNA interference (RNAi) delivery in GBM cells promoted cell cycle arrest and lowered cell proliferation. Upregulation of BAX (BCL2-associated X, apoptosis regulator) induced apoptosis was noted.
Inhibition of MMP14 potentiates the therapeutic effect of temozolomide and radiation in gliomas	Ulasov et al	Cancer Medicine	24156018	2013	MMP 14 knockdown led to reduced tumour growth. Treatment with temozolomide and radiotherapy plus MMP14 blockade drastically improved survival of mice with GBM compared to single therapy. Hence MMP14 inhibition sensitized GBM cells to standard treatment.

Table S5. Tier-2 search results for apoptosis.

Article Title	Authors	Journal	PMID	Year	Outcome
Circular RNA *CDR1as* disrupts the p53/MDM2 complex to inhibit Gliomagenesis	Lou J et al	Molecular cancer	32894144	2020	In vitro and in vivo xenograft mouse model, CDR1as, binding to p53 protein, prevents its ubiquitination by MDM2 and subsequent degradation. This restricts glioma tumorigenesis and cell proliferation by inducing cycle arrest and apoptosis.
RNaseH2A is involved in human gliomagenesis through the regulation of cell proliferation and apoptosis	Dai B et al	Oncology reports.	27176716	2016	shRNA silencing inducing RNaseH2A knowdown blocked tumor cells in G0/G1 phase, thus reducing proliferation, viability and increased apoptosis. RNaseH2A also showed to play an important role in several signaling pathways, included IL-6 and FAS, involved in cell proliferation.
LINC01198 facilitates gliomagenesis through activating PI3K/AKT pathway.	Xie Y, Cheng Y	RNA BIOLOGY VOL. 17, NO. 7, 1040–1052	32378450	2020	Silenced LINC01198 curbed glioma cell proliferation and accelerated cell apoptosis. It also activated the PI3 K/AKT pathway to aggravate glioma progression by targeting PIK3 CA and PTEN and impairing PTEN expression in glioma. Activating PI3 K/AKT signalling, it also exerts oncogenic function in gliomagenesis by regulating PIK3CA and PTEN.
MicroRNA-10b pleiotropically regulates invasion, angiogenicity and apoptosis of tumor cells resembling mesenchymal subtype of glioblastoma multiforme.	Lin J et al	Cellular death and disease	23034333	2012	miR-10b governs invasiveness, angiogenesis, cell growth and apoptosis of U87-2M1 (mesenchymal type) glioma cells – reduced after silencing miR-10b.
Propofol inhibits Wnt signaling and exerts anticancer activity in glioma cells	W. Xu et al	Oncology Letters	29928428	2018	Propofol is a novel potential Wnt signaling inhibitor, suppresses growth and survival of glioma cells in vitro and in vivo. The results indicate a novel mechanism of anticancer activity for propofol and provide novel insights into the signaling pathways regulated by propofol. The results suggested the possibility for the potential use of propofol as a novel agent for the treatment of patients with glioma.
The epidermal growth factor receptor variant type III mutation frequently found	H.M. Kim et al	Cell proliferation: Wiley	33283409	2020	EGFRvIII mutation-induced astrogenesis and massive cell proliferation in a human brain development model. These results provide us new insights into the mechanisms relating EGFRvIII mutation-mediated gliogenesis and gliomagenesis.

in gliomas induces astrogenesis in human cerebral organoids					
Long noncoding RNA LINC00511 induced by SP1 accelerates the glioma progression through targeting miR-124-3p/CCND2 axis	C. Lin et al	Wiley	30973678	2019	LINC00511 was up-regulated in glioma tissues and overexpression positively correlated with the poor prognosis and advanced pathological stages. LINC00511 stimulates proliferation and invasion of glioma cells in vitro while knockdown repressed tumour growth in vivo. LINC00511 upregulated CCND2 expression through competitively sponging with miR-124-3p. Overall, our finding illuminates that LINC00511 is induced by SP1 (transcription factor) and accelerates the glioma progression through targeting miR-124-3p/CCND2 axis, constructing the SP1/LINC00511/miR-124-3p/CCND2 axis.
Sox21 inhibits glioma progression in vivo by forming complexes with Sox2 and stimulating aberrant differentiation	D. Caglayan, E. Lundin, M. Kastemar	International Journal of Cancer	23463365	2013	Transcription factor Sox21 can act as a suppressor gene in gliomagenesis. Induced expression of Sox21 in human glioma cells results in reduced tumor growth and prolonged survival of xenotranplanted mice. Sox21 reduces the stem-cell like properties of the tumor cells, leading to abnormal differentiation, induced apoptosis, and decreased proliferation. The results point to a shift in balance between the counteracting and widely distributed Sox2 and Sox21, revealing the Sox2/Sox21 axis as a target for novel therapy of gliomas.
Sorafenib selectively depletes human glioblastoma tumor-initiating cells from primary cultures	E. Carra, F. Barbieri, D. Marubbi	Cell Cycle (Landes Bioscience)	23324350	2013	Using the sorafenib, the inhibition of two important pathways for GBM biology, MAPK and PI3K/Akt, is obtained leading to the block of proliferation and, most of all, the induction of apoptosis via Mcl-1 inhibition in GBM TICs. Moreover, results demonstrate that sorafenib preferentially affects tumour inducing cells subpopulation, suggests potential use in drug resistance and tumor relapse.
Histone deacetylase inhibitors promote glioma cell death by G2 checkpoint abrogation leading to mitotic catastrophe	M. Cornago et al	Cell Death and Disease (Nature) – Macmillan	25275596	2014	SAHA and VPA, which are more efficient than the chemotherapeutic drug TMZ in killing glioma cells, reduce the expression of proteins involved in processes relevant for tumor growth and survival: G2/M checkpoint gatekeepers, DNA repair mechanisms and proteins involved in mitotic spindle formation and chromosome segregation during mitosis, thus leading to cell death by mitotic catastrophe. These findings support the view that drugs targeting multiple processes are more efficient in killing cancer cells than DNA-damaging agents.
Suppression of autophagy impedes glioblastoma development and induces senescence	N. Gammoh et al	Taylor & Francis	27304681	2016	Autophagy inhibition by RNAi on gliomagenesis in a genetically engineered mouse model in GBN model showed significantly inhibited but not completely ablated. This suggests autophagy is partially inhibited to maintain t homeostatic function of autophagy in normal tissues during pro- longed treatment.

Overexpression of N-myc downstream-regulated gene 1 inhibits human glioma proliferation and invasion via phosphoinositide 3-kinase/AKT pathways	W. Ma et al.	Molecular Medicine Report	25777142	2015	NDRG1 is downregulated in glioma cells, it may play a role in the regulation of growth and invasion of glioma cells. NDRG1 may serve as a potential diagnostic or prognostic marker, and a novel therapeutic target in the treatment of glioma.
Control of glioma cell death and differentiation by PKM2–Oct4 interaction	M. Morfouace et al	Cell Death and Disease	24481450	2014	Dichloroacetate could be a valuable adjuvant therapy for GBM patients, especially in tumors with a large CSC population but needs to be associated with another anticancer drug.
Long noncoding RNA SNHG12 facilitates the tumorigenesis of glioma through miR-101-3p/FOXP1 axis	Yuchen Sun et al	Gene	30098431	2018	Long coding RNA (lncRNAs) have a recognised role in gliomagenesis. This study focuses on lncRNA SNHG12. Knockdown of SNHG12 impaired the proliferation, induced the apoptosis in vitro and, meanwhile, inhibited the tumor growth in vivo
Novel internal regulators and candidate miRNAs within miR-379/miR-656 miRNA cluster can alter cellular phenotype of human glioblastoma	Subhashree Nayak et al	Scientifc reports	29769662	2018	Clustered miRNAs can affect functioning of downstream pathways due to possible coordinated function.<br>Higher expression of the C14MC miRNAs significantly improved glioblastioma prognosis. CRISPR activation of the most efficient internal regulator specifically induced members of the downstream miRNA sub-cluster and apoptosis in glioblastoma cells.
Notch1 is a prognostic factor that is distinctly activated in the classical and proneural subtype of glioblastoma and that promotes glioma cell survival via the NF-κB(p65) pathway	Long Hai et al	Cell death and disease	29410396	2018	Notch1 signaling is a more complex process in the development of numerous cell and tissue types, including gliomagenesis and progression, and is upregulated in glioma-initiating cells. Notch1 and NF-κB(p65) are highly expressed in the classical and proneural subtypes of GBM. DAPT and shRNA targeting Notch1 decreased NF-κB(p65) expression, suppressed cell proliferation, and induced apoptosis of GBM cells in vitro and in vivo

LncRNA HSP90AA1-IT1 promotes gliomas by targeting miR-885-5p-CDK2 pathway	Taihong Gao et al	Oncotarget	29088865	2017	ncRNAs are emerging as important regulators in various types of cancers.<br>Downregulation of HSP90AA1-IT1 in the glioma cell lines significantly suppressed cell viability, proliferation, EMT, invasion and migration in addition to an increase in apoptosis and aberrant cell cycle progression.
Long Noncoding RNA HOXA11-AS Functions as miRNA Sponge to Promote the Glioma Tumorigenesis Through Targeting miR-140-5p	Yan Cui et al	DNA cell biology	28832185	2017	Long noncoding RNAs:<br>Loss-of-function experiments revealed that HOXA11-AS knockdown inhibited the proliferation, induced cell cycle arrest at G0/G1 phase, and enhanced the apoptosis.
HP1α is highly expressed in glioma cells and facilitates cell proliferation and survival	Xianliang Lai et al	Cancer Biomarkers	30372668	2017	Depletion of HP1α (heterochromatin protein 1 α) and SUV39H1 weakens GOS3 and 1321N1 cell proliferation capacity and results in apoptosis of cells. Furthermore, we find that HP1α and H3K9me3 are enriched in the FAS and PUMA promoters, which suggests that upregulated HP1α and H3K9me3 prevent apoptosis by suppressing apoptotic activators. These data indicates that up-regulated HP1α, SUV39H1, and H3K9me3 in glioma cells are functionally associated with glioma pathogenesis and progression
CRNDE Expression Positively Correlates with EGFR Activation and Modulates Glioma Cell Growth	Karrie Mei-Yee Kiang et al	Target oncology	28493025	2017	CRNDE acts as an oncogene that is highly up-regulated in glioma. Expression of CRNDE correlates with EGFR activation. EGF and EGFR tyrosine kinase inhibitor (TKI) enhance and block the up-regulation of CRNDE expression, respectively, suggesting that EGFR signaling may positively regulate CRNDE expression. CRNDE depletion inhibits glioma cell growth both in vitro and in vivo, and is associated with induced cellular apoptosis with decreased Bcl2/Bax ratio.
T11TS repress gliomagenic apoptosis of bone marrow hematopoietic stem cells	Somnath Mondal et al	Journal of cellular physiology	28233371	2017	T11TS (T11 target structure) is also able to diminish the perforin-granzyme B mediated apoptotic verdict of BMHSCs during gliomagenesis. The anti-apoptotic action of T11TS on glioma associated BMHSCs provide a crucial insight into how T11TS exerts its immunomodulatory action against glioma mediated immune devastation

The Inhibition of microRNA-128 on IGF-1-Activating mTOR Signaling Involves in Temozolomide-Induced Glioma Cell Apoptotic Death	Peng-Hsu Chen et al	PLOS one	27893811	2016	miR-128-inhibited mTOR signaling is involved in TMZ-mediated cytotoxicity. Our findings may provide a better understanding of cytotoxic mechanisms of TMZ involved in glioblastoma development.
The role of transcriptional coactivator TAZ in gliomas	Weijie Li et al	Oncotarget	27764783	2016	Transcriptional coactivator with PDZ-binding motif (TAZ) is one of the important downstream effectors of Hippo pathway. When orthotopic glioblastoma mouse model implanted with TAZ knocked down cells, glioma growth was inhibited and survival period was prolonged
Suppression of autophagy impedes glioblastoma development and induces senescence	Noor Gammoh et al	Autophagy	27304681	2016	Autophagy inhibition suppressed the capacity of KRAS-expressing glial cells to form oncogenic colonies or to survive low serum conditions. Molecular analyses revealed that autophagy-inhibited glial cells were unable to maintain active growth signaling under growth-restrictive conditions and were prone to undergo senescence
FRMD6 inhibits human glioblastoma growth and progression by negatively regulating activity of receptor tyrosine kinases	Yin Xu et al	Oncotarget	27661120	2016	FRMD6 is an Ezrin/Radixin/Moesin (ERM) family protein and a human homologue of Drosophila expanded (ex). Results establish a novel function of FRMD6 in inhibiting human GBM growth and progression and uncover a novel mechanism by which FRMD6 exerts its anti-GBM activity.
Functional genomics analyses of RNA-binding proteins reveal the splicing regulator SNRPB as an oncogenic candidate in glioblastoma	Bruna R Correa et al	Genome biology	27287018	2016	NA-binding proteins (RBPs) are master regulators of co- and post-transcriptional events. revealed a prominent oncogenic candidate, SNRPB, which encodes core spliceosome machinery components. To reveal the impact of SNRPB on splicing and gene expression, we performed its knockdown in a GBM cell line followed by RNA sequencing. We found that the affected genes were involved in RNA processing, DNA repair, and chromatin remodeling.

DYT-40, a novel synthetic 2-styryl-5-nitroimidazole derivative, blocks malignant glioblastoma growth and invasion by inhibiting AEG-1 and NF-κB signaling pathways	Meijuan Zou et al	Scientific reports	27251589	2016	Astrocyte elevated gene-1 (AEG-1) has been explored as a novel target for human glioma therapy, thus reflecting its potential contribution to gliomagenesis. This article investigated the effect of DYT-40, a novel synthetic 2-styryl-5-nitroimidazole derivative, on cell growth and invasion in glioblastoma (GBM) and uncovered the underlying mechanisms of this molecule. DYT-40 induces the intrinsic mitochondrial pathway of apoptosis and inhibits the epithelial-mesenchymal transition (EMT) and invasion of GBM cell lines
RNaseH2A is involved in human gliomagenesis through the regulation of cell proliferation and apoptosis	Bin Dai et al	Oncology reports	27176716	2016	RNaseH2A silencing resulted in impaired cell proliferation. clone formation was significantly reduced by RNaseH2A knockdown, which also increased cell apoptosis by approximately 4.5-fold.
IDH1R132H in Neural Stem Cells: Differentiation Impaired by Increased Apoptosis	Kamila Rosiak et al	Plos One	27145078	2016	Expression of IDH1R132H increases apoptosis susceptibility of neural stem cells and their derivatives. Robust apoptosis causes differentiation deficiency of IDH1R132H-expressing cells.
Hugl-1 inhibits glioma cell growth in intracranial model	Xuejiao Liu et al	Journal of neuroonocolgy	26341367	2015	Drosophila lethal giant larvae (lgl) has been reported as a tumor suppressor and could regulate the Drosophila hippo signaling. Human giant larvae-1(Hugl-1), one human homologue of Drosophila lgl, also has been reported to be involved in the development of some human cancers<br>over-expression of Hugl-1 not only inhibited gliomagenesis but also markedly inhibited cell proliferation and promoted the apoptosis of U251 cells in an orthotopic model of nude mice

Table S6. Tier-2 search results for G1/S progression.

Article Title	Authors	Journal	PMID	Year	Outcome
TROAP regulates cell cycle and promotes tumor progression through Wnt/β-Catenin signaling pathway in glioma cells	Zong-Qing Zhao, Xiu-Jie Wu, Yan-Hao Cheng, Yun-Fei Zhou, Xi-Meng Ma, Jian Zhang, Xue-Yuan Heng, Fan Feng	CNS Neuroscience & Therapeutics	34077623	2021	TROAP ((Trophinin Associated Protein) accelerated the progression of gliomagenesis through Wnt/β-Catenin pathway, and TROAP might be considered as a novel target for glioma therapy.
Aberrant nuclear lamina contributes to the malignancy of human gliomas	Shun-qiPei, Xue hui, Wang Xuan, Wang Hao, Huang Huaping, Tao Bing, hua Xie, Aifen Yang, Mengsheng Qiu, Zhou Tan	Journal of Genetics and Genomics	34530169	2021	lamins are proposed to be potential oncogene targets for therapeutic treatments of brain tumors.

Table S7. Tier-2 search resutls for cell cycle progression.

Article Title	Authors	Journal	PMID	Year	Outcome
LRIG2 promotes the proliferation and cell cycle progression of glioblastoma cells in vitro and in vivo through enhancing PDGFRβ signalling	QUNGEN XIAO et al.	INTERNATIONAL JOURNAL OF ONCOLOGY	30015847	2018	Targeting LRIG2, the key oncogenic regulator of multiple RTKs signals, appears to be promising for developing novel therapeutic strategies for the future treatment of GBM.
Znf179 induces differentiation and growth arrest of human primary glioblastoma multiforme in a p53-dependent cell cycle pathway	Kuen-Haur Lee et al.	Nature	28684796	2017	Znf179 induces neuronal differentiation.<br>Decreased expression of Znf179 in glioma brain tumours.<br>Znf179 overexpression enhances differentiation of GBM cells.<br>p53-p21-p27 cell cycle signaling pathways are involved in Znf179-induced differentiation of GBM cells.<br>Glioma patients with higher Znf179 expression levels have longer survival rates
Reciprocal regulation of integrin β4 and KLF4 promotes gliomagenesis through maintaining cancer stem cell traits	Binbin Ma et al.	Journal of Experimental & Clinical Cancer Research	30658712	2019	Existence of a positive feedback loop between KLF4 (Kruppel Like Factor 4) and ITGB4 (Integrin Subunit Beta 4) that promotes GSC self-renewal and gliomagenesis, suggesting that ITGB4 may be a valuable therapeutic target for glioma.
Cyclin-dependent kinase 7 is a therapeutic target in high-grade glioma	SA Greenall et al.	Nature	28504693	2017	CDK7 is crucial for gliomagenesis, CDK7 is a therapeutic target and provide new insight into the cellular processes that are affected by THZ1 and induce antitumor activity.
PTPN2 induced by inflammatory response and oxidative stress contributed to glioma progression	Liquan Wu et al.	Journal of cellular biochemistry	31241223	2019	PTPN2 ((Protein Tyrosine Phosphatase Non-Receptor Type 2))was highly overexpressed in gliomas.<br>PTPN2 was greatly induced by inflammation response in T98G cells.<br>PTPN2 was strongly oxidated and inactivated by ROS.

					PTPN2-deficiency promoted apoptosis in T98G cells<br>PTPN2 was upregulated in glioma cell.
The ATPase Pontin is a key cell cycle regulator by amplifying E2F1 transcription response in glioma	Run Wang et al.	Cell death and disease	33542204	2021	Pontin was overexpressed in gliomas and its higher expression predicted worse prognosis<br>Pontin co-expressed genes were functionally associated with cell cycle control and RNA processing<br>Knockdown of Pontin inhibited cell growth and cell cycle progression in GBM cells<br>The Pontin-affected alternative splicing landscape in GBM cells
Quercetin abrogates IL-6/STAT3 signaling and inhibits glioblastoma cell line growth and migration	Jonathan Michaud-Levesque et al.	Experimental Cell Research	22394507	2012	Quercetin is a potent inhibitor of the IL-6-induced STAT3 signaling pathway in T98G and U87 glioblastoma cells.
LncRNA HSP90AA1-IT1 promotes gliomas by targeting miR-885-5p-CDK2 pathway	Taihong Gao et al.	Oncotarget	29088865	2017	Significantly upregulated in primary glioma.<br>High level was associated with poor prognosis of glioma patients.<br>Affected the viability of glioma cells<br>Knockdown inhibited the migration, invasion and EMT of the glioma cells.
The RNA-binding protein SRSF1 is a key cell cycle regulator via stabilizing NEAT1 in glioma	Xuexia Zhoua et al.	International Journal of Biochemistry and Cell Biology	31200124	2019	NEAT1, whose stability maintained by SRSF1, implicates gliomagenesis by regulating cell cycle. Both SRSF1 and NEAT1 may serve as promising targets for antineoplastic therapies.
CD163, a novel therapeutic target, regulates the proliferation and stemness of glioma cells via casein kinase 2	Taoliang Chen et al.	Oncogene	30258108	2019	CD163 overexpression in human glioma correlated with a poor prognosis<br>CD163 regulates both cell cycle progression and the proliferation of glioma cells<br>Silencing of CD163 inhibits glioma tumorigenicity in vivo<br>CD163 is necessary for maintaining the stemness of primary GSCs
Histologically defined intratumoral sequencing	Antony Prabhu et al.	Neuro-oncology	28541485	2017	Intra-tumoral heterogeneity in glioblastoma is a conserved, predictable consequence to its complex microenvironment, and combinatorial approaches designed to target these unequivocally present tumor biomes may lead to therapeutic gains.

uncovers evolutionary cues into conserved molecular events driving gliomagenesis					
Tumorigenesis and prognostic role of hepatoma-derived growth factor in human gliomas	Shu-Shong Hsu et al.	Journal of Neuro-oncology	22037800	2017	HDGF (hepatoma-derived growth factor) expression is correlated with glioma progression
EZH2 is a negative prognostic factor and exhibits pro-oncogenic activity in glioblastoma	Junxia Zhanga et al.	Cancer Letters	25444902	2015	EZH2 overexpression confers a poor prognosis of GBM patients.<br>EZH2 is an independent prognostic factor in GBM patients.<br>Repression of EZH2 induces cell cycle arrest and inhibits tumor growth in vivo.<br>A feedback loop of EZH2 with β-catenin/TCF4 and STAT3 signaling exists in gliomas.
Comprehensive analysis of the functional microRNA–mRNA regulatory network identifies miRNA signatures associated with glioma malignant progression	Yongsheng Li et al.	Oxford Academic Nucleic acids research	24194606	2013	hsa-miR-524-5p and hsa-miR-628-5p exhibited dominant regulatory activities. Therefore, network-based biomarkers are expected to be more effective and provide deep insights into the molecular mechanism of glioma malignant progression
Ehd3, a regulator of vesicular trafficking, is silenced in gliomas and functions as a tumor suppressor by controlling cell cycle arrest and apoptosis	Sahiti Chukkapalli et al.	Oxford Academic Carcinogenesis	24306026	2014	Loss of EHD3 expression in gliomas, including low-grade astrocytomas, suggesting that this is an early event in gliomagenesis<br>Ehd3 functions as a tumor suppressor gene and loss of its expression is a very common event in gliomas.

Table S8. Tier-3 search results for theranostics in GBM.

Article Title	Authors	Journal	PMID	Year	Outcome
Dramatic response of BRAF V600E-mutant epithelioid glioblastoma to combination therapy with BRAF and MEK inhibitor: establishment and xenograft of a cell line to predict clinical efficacy	Yu Kanemaru et al.	Acta Neuropathologica Communications	31345255	2019	*BRAF* V600E mutation, associated with aggressive epithelioid GBMs responds well to combined treatment with BRAF and MEK inhibitors.
miR-504 suppresses mesenchymal phenotype of glioblastoma by directly targeting the FZD7-mediated Wnt–β- catenin pathway	Qiang Liu et al.	Journal of Experimental & Clinical Cancer Research	31419987	2019	miR-504 suppresses GBM mesenchymal transition by inhibiting FZD7 expression and negatively regulating Wnt–β-catenin pathway.
The miR-26a/AP-2α/Nanog signaling axis mediates stem cell self-renewal and temozolomide resistance in glioma	Wenhuan Huang et al.	Theranostics	31534499	2019	AP-2α reduces the stemness and TMZ resistance of glioma by inhibiting the Nanog/Sox2/CD133 axis and IL6/STAT3 signalling pathways.
New insights for precision treatment of glioblastoma from analysis of single-cell lncRNA expression	Meng et al	J Cancer Res Clin Oncol.	33693962	2021	Single cell long non-coding RNA (lncRNA) expression used to re-profile GBM with evidence from TCGA, more representative of GBM heterogenicity. 3 patient populations identified, with 4 cell subtypes.<br>Population 1: demonstrated cell subtypes 2 and 3 with lnc markers: LINC00461 and LINC00339 involved in cell proliferation. LINC00339 is blocked by avasimibe and inhibits cell tumourgencity<br>Population 2: recognised by MALAT1 it confers chemotherapy resistance by blocking miRNA-203 to enhance thymidylate synthase, improves response to temozolomide.<br>Population 3: best survival prognosis, it expressed MEG3 – this blocks GBM growth and apoptosis. MEG3 is associated with lower WHO grade and higher Karnofsky score.

Predictive MGMT status in a homogeneous cohort of IDH wildtype glioblastoma Patients	Josefine Radke et al.	Acta Neuropathologica Communications	31167648	2019	Methylation status of MGMT can be classified as high (>20%), low (10-20%) and unmethylated (<20%) where high methylation levels positively correlate with progression-free and overall survival.
Genomic alterations predictive of response to radiosurgery in recurrent IDH-WT glioblastoma	Dono et al.	J Neurooncol.	33492602	2021	Recurrent IDH GBM patients with PTEN mutations had a lengthier progression free survival and overall survival in response to SRS. This prognostic marker can detect those most likely to response to SRS.
(68)Ga-Pentixafor-PET/CT for Imaging of Chemokine Receptor 4 Expression in Glioblastoma.	Lapa C, Lückerath K, Kleinlein I, et al.	Theranostics	26909116	2016	Chemokine receptor-4 (CXCR4) is overexpressed in GBM and is associated with poor survival. Direct imaging of CXCR-4 with PET or CT scans with a CXCR4 ligand 68Ga-Pentixafor. This method to quantify CXCR-4 in GBM patients in the future and to select patient who may benefit from CXCR4-directed therapy.
177Lu-/68Ga-PSMA Theranostics in Recurrent Glioblastoma Multiforme: Proof of Concept (FAV)	Kumar et al.	Clinical Nuclear Medicine	32558721	2020	Case report of 37 y.o male with MRI confirmed Lt temporal GBM. Exploratory Ga-PSMA PET/CT demonstrated PSMA expression in the recurrent lesion. After 3 cycles of Lu-PSMA-617, Ga-PSMA PET/CT showed significant reduction in PSMA uptake and regression in size of lesion on MRI with improvement in patient's symptoms and performance status.
Multiparametric magnetic resonance imaging in the assessment of anti-EGFRvIII chimeric antigen receptor T cell therapy in patients with recurrent glioblastoma	Wang et al.	British Journal of Cancer	30478409	2019	EGFRvIII targeted chimeric antigen receptor T (CAR-T) cell therapy has recently been reported for treating glioblastomas (GBMs). This paper showed a group of 10 patients who underwent multiparametric MRI at baseline, 1, 2 and 3 months after CAR-T therapy. Logistic regression model derived progression probabilities (PP) using imaging parameters were used to assess treatment response. Results indicate that multiparametric MRI may be helpful in monitoring CAR-T related early therapeutic changes in GBM patients.

Table S9. Tier-4 search results for vaccines in GBM.

Article Title	Authors	Journal	PMID	Year	Outcome
A Phase I Study of Autologous Dendritic Cell Vaccine Pulsed with Allogeneic Stem-like Cell Line Lysate in Patients with Newly Diagnosed or Recurrent Glioblastoma	Hu et al.	Clinical Cancer Research	34862245	2021	Autologous dendritic cell vaccines pulsed with lysate originating from GBM stem-like cell lines was safe and without significant side effects.
A phase I study of the WT2725 dosing emulsion in patients with advanced malignancies	Fu et al.	Scientific Reports	34785698	2021	Wilms' tumor gene 1 (WT1) overexpressed GBM derived oligopeptide vaccine WT2725 produced significant immune response by inducing cytotoxic T-lymphocytes leading to cell lysis and attenuation cell proliferation. Third of patients (n=20) survived >1 year. Progression free survival 59 days and overall survival 309 days.
Immunogenic Cell Death Induced by Chemoradiotherapy of Novel pH-Sensitive Cargo-Loaded Polymersomes in Glioblastoma	He et al.	International journal Nandomedicine	34712045	2021	Angiopep-2-conjugated pH-sensitive polymersomes (Au-DOX@PO-ANG) a nanoparticle that crosses the blood brain barrier. Au-DOX@PO-ANG in conjunction with radiotherapy promotes GBM cells death which stimulates dendritic cells maturation via DAMPs and causes Tumour antigen specific T cell to immunogenic cell death.
Identification of Tumor Antigens and Immune Landscape in Glioblastoma for mRNA Vaccine Development	Ye at al.	Frontiers Genetics	34527020	2021	Prognostic mRNA antigens: ARPC1B and HK3 identified from TCGA were positively correlated with antigen presenting cells in GBMs validating role in immune response. Immune subtype 2 had "pro-inflammatory features" better response to vaccines.
Distinct difference in tumor-infiltrating immune cells between Wilms' tumor gene 1 peptide vaccine and anti-programmed cell death-1 antibody therapies	Yokota et al.	Neurooncology Advances	34355173	2021	Wilms' tumor gene 1 (WT1) peptide vaccine and anti-programmed cell death-1 (anti-PD-1) combination improved mice survival compared with monotherapy.
MR1 Tetramer-Based Artificial APCs Expand MAIT Cells from Human	Priya et al.	Immunohorizons	34172533	2021	Mucosal-associated invariant T cells (MAIT) amplified from human peripheral blood using artificial MR1 tetramer APCs retained cytotoxic activity against GBM cells.

Peripheral Blood That Effectively Kill Glioblastoma Cells					highlights role of MAIT.
BRD4 inhibition boosts the therapeutic effects of epidermal growth factor receptor-targeted chimeric antigen receptor T cells in glioblastoma	Xia et al.	Molecular Therapy: the journal of American gene society	34058385	2021	Epigenetic regulator BRD4 inhibitor JQ1 prevented stimulation of immunosuppressive genes to augment EGFR CAR-T (chimeric antigen receptor T) cell action in attenuating growth and metastasis in GBM.
90K predicts the prognosis of glioma patients and enhances tumor lysate-pulsed DC vaccine for immunotherapy of GBM in vitro	Zeng et al.	Aging (Albany NY)	33686953	2021	90K (tumour associated antigen) potentiated the effect of apoptosis U251-Dendritic cell vaccine to kill GBM cells via increasing cytotoxic T cell activity.
New generation of DNA-based immunotherapy induces a potent immune response and increases the survival in different tumor models	Lopes et al.	Journal for immunotherapy of cancer	33795383	2021	Viral glycoprotein employed as a carrier of T cell tumor epitopes, prior to tumour debulk results in longer remission and survival. It enhanced cytotoxic T cell activity against GBM cells.
Malignant Glioma Therapy by Vaccination with Irradiated C6 Cell-Derived Microvesicles Promotes an Antitumoral Immune Response	Benjamín Pineda et al.	Molecular Therapy	31204210	2019	Vaccination with irradiated cell-derived microvesicles led to reduction of tumour volume and increased immune cell infiltration of the implanted tumour tissue.
Impaired circulating myeloid CD1c+ dendritic cell function in human glioblastoma is restored by	Jason Adhikaree et al.	Oncoimmunology	31143512	2019	Inhibition of p38 restored GBM related dendritic cell dysfunction by restoring IL-12:IL-10 ratio leading to enhanced T-cell proliferation and interferon-gamma (IFNγ) production.

p38 inhibition – implications for the next generation of DC vaccines					
A Randomized Double-Blind Placebo-Controlled Phase II Trial of Dendritic Cell Vaccine ICT-107 in Newly Diagnosed Patients with Glioblastoma	Patrick Y. Wen et al	Clinical Cancer Research	31320597	2019	Immunization with ICT-107, autologous DC pulsed with GBM-associated antigens, significantly improved progression-free survival with maintenance of quality of life.
Immunological analysis of phase II glioblastoma dendritic cell vaccine (Audencel) trial: immune system characteristics influence outcome and Audencel up-regulates Th1-related immunovariables	Friedrich Erhart et al.	Acta Neuropathologica Communications	30518425	2018	DC immunotherapy against GBM stimulates the immune system especially Th-1 related immunovariables, however these did not correlate with positive clinical outcomes regarding survival.
Neoantigen vaccine generates intratumoral T cell responses in phase Ib glioblastoma trial	Derin B Keskin et al.	Nature	30568305	2019	Personalized neoantigen -targeting vaccination led to generation of polyfunctional neoantigen-specific CD4+ and CD8+ T cell responses as well as increased T-cell infiltration of the tumour.
Phase I/II trial testing safety and immunogenicity of the multipeptide IMA950/poly-ICLC vaccine in newly diagnosed adult malignant astrocytoma patients	Denis Migliorini et al.	Neuro-Oncology	30753611	2019	Combining IMA950 peptide vaccine with poly-ICLC led to multipeptide CD8 and sustained T-helper 1 CD4 T-cell responses.
Actively personalized vaccination trial for newly diagnosed glioblastoma	Hilf N et al.	Nature.	30568303	2019	Phase I trial GAPVAC-101 of the Glioma Actively Personalized Vaccine Consortium (GAPVAC), where they used personalised vaccines with unmutated antigen sand neoepitopes as therapy for newly diagnosed gliomas.15 patients with GBM positive for human leukocyte antigen (HLA)-A*02:01 or HLAA*24:02 were treated with a vaccine

					(APVAC1) derived from unmutated antigens and then followed by treatment with APVAC2 (targets neoepitopes).<br>This treatment showed excellent safety and strong immunogenicity i.e. responses of central memory CD8+ T cells and CD4+ T cell responses of T helper 1 type against predicted neoepitopes.
Immunological analysis of phase II glioblastoma dendritic cell vaccine (Audencel) trial: immune system characteristics influence outcome and Audencel up-regulates Th1-related immunovariables.	Erhart F et al.	Acta Neuropathol Commun.	30518425	2018	Audencel- a DC based cellular cancer immunotherapy against GBM. They found patient with GBM with favourable anti-tumour characteristics showed longer survival rates. They deduced that even though Audencel's previous failure to induce a significant clinical response, it did have an effect on the immune system and its effect depends on the state of the immune system as it seems to stimulate immune systems of patients.
A randomized, double-blind, phase III trial of personalized peptide vaccination for recurrent glioblastoma	Narita Y et al.	Neuro Oncol.	30500939	2019	A Phase III trial of personalized peptide vaccination (PPV) for human leukocyte antigen (HLA)-A24+ recurrent glioblastoma. Trial failed to meet primary or secondary endpoints.
Audencel Immunotherapy Based on Dendritic Cells Has No Effect on Overall and Progression-Free Survival in Newly Diagnosed Glioblastoma: A Phase II Randomized Trial.	Buchroithner J et al.	Cancers (Basel).	30301187	2018	DC (antigen-presenting cells) were used in multicentric randomized open-label phase II study by tumor lysate-charged autologous DCs (Audencel). They found no difference in progression free survival in control and vaccine group.
Treatment of Human Glioblastoma with a Live Attenuated Zika Virus Vaccine Candidate	Chen Q et al.	mBio.	30228241	2018	Paper explored the use of live attenuated ZIKV vaccine candidate (ZIKV-LAV) as a GBM treatment. They found it has greater neurovirulence that of the licensed Japanese encephalitis virus LAV 14-14-2 and showed no neurological abnormalities in the injected mice. Therefore, there may be room for further study of ZIKV-LAV for GBM treatment.
Molecular subgroups and B7-H4 expression levels predict responses to den-	Yao Y et al.	Cancer Immunol Immunother.	30159779	2018	DC based vaccination in a double-blind, placebo-controlled phase II clinical trial. Participants were separated into different molecular subgroups based on the mutation status of IDH1/2 and telomerase reverse transcriptase (TERT). Their results showed IDH1 wild

dritic cell vaccines in glioblastoma: an exploratory randomized phase II clinical trial.					type TERT mutation status and low B7-H4 expression identified subgroups of GBM patients more responsive to GSC DCV-based specific active-immunotherapy.
A Dendritic Cell-Targeted Adenoviral Vector Facilitates Adaptive Immune Response Against Human Glioma Antigen (CMV-IE) and Prolongs Survival in a Human Glioma Tumor Model.	Kim JW et al..	Neurotherapeutics.	30027430	2018	DC based immunization approach has the significant therapeutic potential for patients with high-grade gliomas
Predictors of Response to Autologous Dendritic Cell Therapy in Glioblastoma Multiforme.	Jan CI et al..	Front Immunol.	29910795	2018	27 patients who received DC vaccines post surgically along with conventional treatment with radiotherapy and temozolamide and compared them with 20 patient with the conventional treatment for GBM without DC vaccines.<br>They found younger age, gross resection of tumour, tumour infiltrating lymphocytes or peripheral blood mononuclear cells with lower PD-1+/CD8+ ratio were predictor of longer survival rates.
Tumor lysate-loaded Bacterial Ghosts as a tool for optimized production of therapeutic dendritic cell-based cancer vaccines.	Dobrovolskienė N et al.	Vaccine.	29895501	2018	Enhanced DC vaccines effects by using bacterial ghosts as a delivery system. Bacterial ghosts are empty envelopes of gram negative bacteria. They found they were an excellent method of induction of immunogenic DC maturation and loading with tumour antigens. They compared it to the convention method of lipopolysaccharide (LPS) plus interferon-γ cocktail. They found the Bacterial ghost method had superior CD8+ T cell activation
Zika virus-based immunotherapy enhances long-term survival of rodents with brain tumors through upregulation of memory T-cells	Andrew T Crane et al.	Plos ONE	33002018	2020	Zika virus (ZIKV) is known to exhibit tropism for brain tumour cells and has been demonstrated to have an oncolytic effect. Mice bearing malignant intracranial GL261 tumors were subcutaneously vaccinated with irradiated GL261 cells previously infected with the ZIKV. Mice also received intracranial injections of live ZIKV, irradiation attenuated ZIKV, or irradiated GL261 cells previously infected with ZIKV. Long-term survivors were rechallenged with a second intracranial tumor to examine their immune response and look for the establishment of protective memory T-cells.

					Subcutaneous vaccination plus intracranial irradiation attenuated ZIKV or intracranial irradiated GL261 cells previously infected with ZIKV exhibited the greatest extensions to overall survival
Activation of 4-1BBL+ B cells with CD40 agonism and IFNγ elicits potent immunity against glioblastoma	Catalina Lee-Chang et al.	Journal of experimental medicine	32991668	2021	B cell-based vaccine (BVax) that consists of 4-1BBL+ B cells activated with CD40 agonism and IFNγ stimulation. Bvax migrates to key secondary lymphoid organs and is proficient at antigen cross-presentation, which promotes both the survival and the functionality of CD8+ T cells. A combination of radiation, BVax, and PD-L1 blockade conferred tumor eradication in 80% of treated tumor-bearing animals. elicited immunological memory that prevented the growth of new tumors upon subsequent reinjection in cured mice. GBM patient-derived BVax was successful in activating autologous CD8+ T cells; these T cells showed a strong ability to kill autologous glioma cells.
Peptide Vaccine Combined Adjuvants Modulate Anti-tumor Effects of Radiation in Glioblastoma Mouse Model	Thi-Anh-Thuy Tran et al.	Frontiers immunology	32733437	2020	Immunotherapy comprising a branched multipeptide plus PADRE, poly-ICLC, and anti-PD1 could potentially enhance the anti-tumor effects of radiotherapy in a glioblastoma mouse model
CD133 mRNA-Loaded Dendritic Cell Vaccination Abrogates Glioma Stem Cell Propagation in Humanized Glioblastoma Mouse Model	Angelique Sao-Mai Sy Do et al.	Molecular therapy oncolytics	32728617	2020	CD133 is a cell surface antigen that is present on glioma stem cells and has been used to prospectively isolate glioma stem cells. confirmed the robust simultaneous activation of CD8- and CD4-positive T cells by dendritic cell vaccination with modified CD133 mRNA leading to a potent and long-lived immune response, with subsequent abrogation of CD133-positive glioma stem cell propagation and tumor growth.
Once, Twice, Three Times a Finding: Reproducibility of Dendritic Cell Vac-	Kristen A Batich et al.	Clinical cancer research	32719000	2020	Conducted three separate clinical trials utilizing cytomegalovirus (CMV)-specific DC vaccines in patients with newly diagnosed glioblastoma whereby each follow-up study had nearly doubled in sample size. Follow-up data from the first blinded, randomized phase II clinical trial (NCT00639639) revealed that nearly one third of this cohort is without tumor recurrence at 5 years from diagnosis. A second clinical trial (NCT00639639) resulted in a 36% survival rate at 5 years from diagnosis. Results of the first two-arm trial

cine Trials Targeting Cytomegalovirus in Glioblastoma					(NCT00639639) showed increased migration of the DC vaccine to draining lymph nodes, and this increased migration has been recapitulated in our larger confirmatory clinical study (NCT02366728). We have now observed that nearly one third of the glioblastoma study patient population receiving CMV-specific DC vaccines results in exceptional long-term survivors.
Expansion of effector and memory T cells is associated with increased survival in recurrent glioblastomas treated with dendritic cell immunotherapy	Marica Eoli et al.	Neuro-oncology advances	32642658	2019	In patients with GBM recurrence the immune-mediated effects of DC immunotherapy loaded with autologous tumor lysate combined with temozolomide (TMZ) or tetanus toxoid (TT). They found that TT preconditioning of the vaccine site and lack of TMZ could contribute to the efficacy of DC immunotherapy by inducing an effector response, memory, and helper T-cell generation.
Feasibility of dendritic cell-based vaccine against glioblastoma by using cytoplasmic transduction peptide (CTP)-fused protein antigens combined with anti-PD1	Young-Hee Kim et al.	Human vaccines and immunotherapies	32401608	2020	DC immunotherapy was pulsed with CTP-fused protein antigens combined with programmed cell death protein 1 blockade (anti-PD1). Using CTP-fused protein antigens for DC vaccine preparation along with PD1 blockade could be an effective immunotherapy strategy for GBM.
Clinical and histopathological analyses of VEGF receptors peptide vaccine in patients with primary glioblastoma - a case series	Ryota Tamura et al.	BMC cancer	32164575	2020	VEGFR1 and 2 vaccination may have a preliminary synergistic effect when administered with TMZ. The limitation of the present study was the paucity of the number of the samples. Further studies involving more patients are warranted to confirm the findings of this study.

Treatment of an aggressive orthotopic murine glioblastoma model with combination checkpoint blockade and a multivalent neoantigen vaccine	Connor J Liu et al.	Neuro oncology	32133512	2020	Endogenous neoantigen specific CD8+ T cells within an αPD-L1 resistant murine GBM and show that neoantigen vaccination significantly augments survival benefit in combination with αPD-L1 treatment.
Tumor-associated antigen-based personalized dendritic cell vaccine in solid tumor patients	Qian-Ting Wang et al.	Cancer immunology, immunotherapy	32078016	2020	Tumor-associated antigens (TAAs) have been tested in various clinical trials. Tumor samples from patients were first analyzed to identify overexpressed TAAs. Autologous DCs were then transfected with pre-manufactured mRNAs encoding the full-length TAAs. A total of ten patients were treated with DC vaccines transfected with personalized TAA panels containing 3-13 different TAAs. The treated patients were associated with favorable overall survival when compared to patients who received standard treatment in the same institution.
Convection-enhanced delivery of temozolomide and whole cell tumor immunizations in GL261 and KR158 experimental mouse gliomas	Julio Enríquez Pérez et al.	BMC cancer research	33109586	2017	Immunotherapy synergized with CED-TMZ in the GL261 model and cured animals in the KR158 model. Single bolus administration of TMZ was effective with a narrower therapeutic window than CED-TMZ. Combined CED-TMZ and immunotherapy led to an increase in the intratumoral influx of T-cells.
Alpha-type-1 Polarized Dendritic Cell-based Vaccination in Newly Diag-	Koichi Mitsuya et al.	Anti-cancer research	33109586	2020	Sixteen newly diagnosed patients with high-grade gliomas were enrolled in a phase II study of the α-type-1 DC vaccine. DCs obtained from the culture of enriched monocytes in the presence of a cytokine cocktail, were pulsed with a cocktail of 5 synthetic peptides and cryopreserved until injection into patients.

nosed High-grade Glioma: A Phase II Clinical Trial					Peptide-cocktail-pulsed α-type-1 DC vaccines have a potential therapeutic effect on survival when used in combination with the standard regimen, which is partly based on IL-12-IFN-γ-mediated T-cell activation.
First results on survival from a large Phase 3 clinical trial of an autologous dendritic cell vaccine in newly diagnosed glioblastoma	Liau LM et al.	J Transl Med.	29843811	2018	Phase 3 trial was conducted and DC vaccines were added to the conventional method of treating GBMs with resection and temozolomide. They found it was safe to add dendritic cell vaccines and could potentially elongate survival.
Heat shock protein peptide complex-96 vaccination for newly diagnosed glioblastoma: a phase I, single-arm trial.	Ji N, Zhang et al.	JCI Insight.	29769450	2018	Single-arm, phase I study in which adult patients were vaccinated with HSPPC-96 (Heat shock protein peptide complex-96) in combination with the standard treatment for newly diagnosed GBM after surgical resection. They found that it was safe treatment with increased efficacy of GBM treatment.
Survival gain in glioblastoma patients treated with dendritic cell immunotherapy is associated with increased NK but not CD8+ T cell activation in the presence of adjuvant temozolomide.	Pellegatta S et al.	Oncoimmunology.	29632727	2018	Two-stage phase II study where patient received dendritic cell vaccine after standard therapy with temozolomide. Dendritic cell vaccine showed a activation of NK cells which translated to a prolonged survival but did not activate CD8+ T cells.
Circulating CD9+/GFAP+/survivin+ exosomes in malignant glioma patients following survivin vaccination.	Galbo PM Jr et al.	Oncotarget.	29383115	2017	Low levels of CD9+/GFAP+/SVN+ exosomes in cancer free individuals although CD9+/GFAP+ exosomes were present in small numbers. They demonstrated that patients with malignant gliomas had CD9+/GFAP+/SVN+ and CD9+/SVN+ exosomes. Flow cytometry to was used for quantitative measurements of these exosomes who had undergone treatment with anti-survivin vaccine (SurVaxM).<br>They found that patients with longer time to tumour progression showed a decrease in CD9+/SVN+ and CD9+/GFAP+/SVN+ exosomes following survivin vaccination as compared to those with shorter time to tumour progression exhibited increased exosomes regardless of SurVaxM.

Dendritic Cells Enhance Polyfunctionality of Adoptively Transferred T Cells That Target Cytomegalovirus in Glioblastoma.	Reap EA et al.	Cancer Res.	29093005	2018	A randomised trial was conducted in which some patients received CMV pp65-specific T cells with CMV-DC vaccination (CMV-ATCT-DC) and were compared to patients who had received saline (CMV-ATCT-saline).<br>Patients who received CMV-ATCT-DC vaccination experienced a significant increase in the overall frequencies of IFNγ+, TNFα+, and CCL3+ polyfunctional, CMV-specific CD8+ T cells which translated to longer overall survival
Co-delivery of tumor-derived exosomes with alpha-galactosylceramide on dendritic cell-based immunotherapy for glioblastoma.	Liu H et al.	Cancer Lett.	28947140	2017	Tumor-associated antigen specific Dendritic cells with an invariant natural killer T cell adjuvant was used in orthotopic rasts with GBM who were vaccinated with tumour-derived exosomes and α-galactosylceramide -pulsed dendritic cells. This produced robust activation and proliferation of tumour-specific cytotoxic T lymphocytes.
Rindopepimut with temozolomide for patients with newly diagnosed, EGFRvIII-expressing glioblastoma (ACT IV): a randomised, double-blind, international phase 3 trial.	Weller M et al.	Lancet Oncol.	28844499	2017	Rindopepimut is a vaccine targeting the EGFR deletion mutation EGFRvIII which the authors used in a randomised, double-blind, international phase 3 trial to explore if the addition of rindopepimut to chemotherapy is able to improve survival in patients with EGFRvIII-positive glioblastoma. They found it did not increase survival in newly diagnosed GBM.
A phase II trial of autologous dendritic cell vaccination and radiochemotherapy following fluorescence-guided surgery in newly diagnosed glioblastoma patients.	Inogés S et al.	J Transl Med.	28499389	2017	Phase-II clinical trial autologous dendritic cell vaccination in patients with newly diagnosed patients GBM and found it was a safe and effective treatment. Overall survival was better in patients with methylated MGMT promoter.
Exosomes isolated from plasma of glioma patients enrolled in a vaccination trial reflect antitumor immune activity and might predict survival	Laurent Muller et al.	Oncoimmunology	26155415	2015	Protein and mRNA expression levels for immune-related genes in plasma exosomes were useful in evaluating glioma patients' response to vaccination therapy.

Dendritic cell immunotherapy for brain tumors	Joseph P et al.	Journal of Neuro-Oncology	26037466	2015	DC vaccines have shown efficacy in multiple pre-clinical studies and Phase I/II clinical trials for<br>gliomas. While acid-eluted peptide and glioma-associated<br>peptide preparations have shown some clinical benefit
Effective Treatment of Established GL261 Murine Gliomas through Picornavirus Vaccination-Enhanced Tumor Antigen-Specific CD8+ T Cell Responses	Danielle N Renner et al.	PLOS one	25933216	2015	GL261-Quad gliomas elicit MHC class I restricted CD8+ T cell responses against tumor-specific antigen. Vaccination with an engineered picornavirus significantly increases levels of tumor antigen-specific CD8+ T cells infiltrating the CNS.<br>Enhanced CD8+ T cell responses toward GL261-Quad gliomas are accompanied by delayed tumor outgrowth and prolonged survival. Functional cytotoxic activity of CD8+ T cells is required for picornavirus vaccination efficacy against GL261-Quad gliomas
First clinical results of a personalized immunotherapeutic vaccine against recurrent, incompletely resected, treatment-resistant glioblastoma multiforme (GBM) tumors, based on combined allo- and auto-immune tumor reactivity	Virgil E.J et al.	Vaccine	25865468	2015	Gliovac (or ERC 1671) composed of autologous antigens, derived from the patient's surgically removed tumor tissue, administered together with allogeneic antigens from glioma tissue resected from other GBM patients.<br>Gliovac administration in patients that have failed standard of care therapies showed minimal toxicity and enhanced overall survival.<br>Six-month (26 weeks) survival for the nine Gliovac patients was 100% versus 33% in control group. At week 40, the published overall survival was 10% if recurrent, reoperated patients were not treated. In the Gliovac treated group, the survival at 40 weeks was 77%
Use of ERC-1671 Vaccine in a Patient with Recurrent Glioblastoma Multiforme after Progression during Bevacizumab Therapy: First Published Report	Daniela A Bota et al.	The permanente journal	PMC4403580	2015	Patients survived for ten months after the vaccine administration without any other adjuvant therapy and died of complications related to his previous chemotherapies. The tissues collected after two vaccination cycles and at the time of death showed a robust immune response and no viable tumor. Preliminary data strongly indicate that ERC-1671 could be effective in the treatment of progressive malignant gliomas
Wilms tumor 1 peptide vaccination combined with temozolomide against newly diagnosed glioblastoma: safety and	Naoya Hashimot et al.	Cancer Immunology, Immunotherapy	25772149	2015	Wilms tumor 1 peptide vaccination.1 week after the end of combined concurrent radio/temozolomide therapy, and administration was continued once per week for 7 weeks.A progression-free survival from histological diagnosis of 5.2–49.1 months

impact on immunological response					
Tetanus toxoid and CCL3 improve dendritic cell vaccines in mice and glio-blastoma patients	Duane Aet al.	Nature	PMC4510871	2015	Modulation of CMV-specific DCs with Td pre-conditioning increases their migratory capacity and may improve clinical outcomes in patients with GBM. Corroborating studies in mice support these claims and underline CCL3 as a novel and important mediator of increased DC migration to VDLNs guiding naive CD81 localization to sites of DC–CD41 interactions in lymph nodes.
OX40 ligand expressed in glioblastoma modulates adaptive immunity depending on the microenvironment: a clue for successful immunotherapy	Ichiyo Shibahara et al.	Molecular Cancer	PMC4339477	2015	OX40L mRNA was expressed in glioblastoma specimens and higher levels were associated with prolonged progression-free survival of patients with glioblastoma, who had undergone gross total resection.<br>OX40 agonist antibody together with glioma cell lysates elicited stronger antitumor immunity and prolonged the survival of mice bearing glioma or glioma-initiating cell-like cells.<br>OX40 triggering activated regulatory T cells cultured under hypoxia led to the induction of the immunosuppressive cytokine IL10.
A phase II, multicenter trial of rindopepimut (CDX-110) in newly diagnosed glioblastoma: the ACT III study	James Schuster et al,	Oxford Academic Neuro-oncology	25586468	2015	Progression-free survival at 5.5 months (~8.5 mo from diagnosis) was 66%.<br>Median overall survival was 21.8 months, and 36-month overall survival was 26%.<br>Extended rindopepimut vaccination (up to 3.5+ years) was well tolerated.<br>Anti-EGFRvIII antibody titers increased ≥4-fold in 85% of patients and increased with duration of treatment.<br>EGFRvIII was eliminated in 4/6 (67%) tumor samples obtained after >3 months of therapy.
Enhancement of glioma-specific immunity in mice by "NOBEL", an insulin-like growth factor 1 receptor antisense oligodeoxynucleotide	Mélanie Morin-Brureau et al.	Cancer Immunology, Immunotherapy	25579379	2015	In vitro treatment of GL261 with NOBEL IGF-1R/ AS-ODN inhibits IGF-1R expression with minimal<br>Cytotoxicity. NOBEL is immunostimulatory but only effective in preventing tumor development when co administered with GL261 cell
Human CD14+ cells loaded with Paclitaxel inhibit in vitro cell proliferation of glioblastoma	Arianna Bonomi et al.	Cytotherapy	25457277	2015	Both CD14+ and DCs were able to incorporate PTX and release the drug in the CM in a time-dependent manner (maximal release over 24 h). The addition of CM from CD14+ and DCs loaded with PTX strongly inhibits proliferation of U87 MG cells.

					Peripheral blood-derived CD14+ and DCs, in addition to their application for immunotherapy for GB, could also be used to delivery anti-cancer drugs, such as PTX, to kill GB cells.
Glioblastoma exosomes and IGF-1R/AS-ODN are immunogenic stimuli in a translational research immunotherapy paradigm	Larry A Harshyne et al.	Cancer Immunology, Immunotherapy	25391690	2015	Vaccine consisting of Lucite diffusion chambers containing irradiated autologous tumor cells pre-treated with an antisense oligodeoxynucleotide (AS-ODN) directed against the insulin-like growth factor type 1 receptor was found to elicit positive clinical responses in 8/12 patients when implanted in the rectus sheath for 24 h<br>Release of AS-ODN together with selective release of exosomes from glioblastoma cells implanted in chambers may drive the therapeutic effect seen in the pilot vaccine trial.
Dendritic cell vaccination combined with temozolomide retreatment: results of a phase I trial in patients with recurrent glioblastoma multiforme	Martin K Hunn et al.	Journal of Neuro-oncology	25366363	2015	GBM patients relapsing after TMZ with a combination of surgery, autologous DC-based vaccines<br>and further TMZ is safe but is hampered by uncertainty in<br>achieving the required vaccine yield.
Phase I/IIa trial of fractionated radiotherapy, temozolomide, and autologous formalin-fixed tumor vaccine for newly diagnosed glioblastoma	Eiichi Ishikawa et al.,	Journal of Neurosurgery	24995786	2015	This treatment regimen was well tolerated by all patients.<br>The percentage of patients with progression-free survival (PFS) ≥ 24 months was 33%.<br>The median PFS, median overall survival (OS), and the actuarial 2- and 3-year survival rates of the 24 patients were 8.2 months, 22.2 months, 47%, and 38%,
Immunomodulation Mediated by Anti-angiogenic Therapy Improves CD8 T Cell Immunity Against Experimental Glioma	Malo CS et al.	Frontiers in Oncology	30211113	2018	VEGF-Trap treatment has anti-angiogenic effect, normalizing the vasculature and enhance tumor antigen-specific CD8T cell response.
Molecular subgroups and B7-H4 expression levels predict responses to dendritic cell vaccines in glioblastoma: an exploratory	Yao Y et al.al	Cancer Immunol Immunother.	30159779	2018	DC based vaccination increases overall survival and progression-free survival in glioblastoma patients, and also increases plasma levels of cytokines CCL22 and IFN-γ compared with placebo.

randomized phase II clinical trial.					
Phase II study of ERC1671 plus bevacizumab versus bevacizumab plus placebo in recurrent glioblastoma: interim results and correlations with CD4+ T-lymphocyte counts.	Bota DA et al.	CNS Oncol	30157683	2018	The combination of the novel immunotherapy ERC1671 and bevacizumab had good overall survival rates
Therapeutic Immunization against Glioblastoma.	Schijns VEJC et al.	Int J Mol Sci.	30150597	2018	GLIOVAC/ERC1671 therapy in individual late-stage relapsing patients had significantly increase of overall survival rates of 6 months, with no severe adverse effects.
A Dendritic Cell-Targeted Adenoviral Vector Facilitates Adaptive Immune Response Against Human Glioma Antigen (CMV-IE) and Prolongs Survival in a Human Glioma Tumor Model.	Kim JW et al.	Neurotherapeutics	30027430	2018	A novel adenovirus (Ad5scFvDEC205FF) is used as a viral vector that specifically infects dendritic cells and can be delivered systemically, activating effector T cells and leading to a robust and long-lasting immune response against the tumor. It's a combination between virotherapy and immunotherapy
Dendritic cells cross-talk with tumour antigen-specific CD8+ T cells, Vγ9$\gamma\delta$T cells and Vα24NKT cells in patients with glioblastoma multiforme and in healthy donors.	Eiraku Y et al.	Clin Exp Immunol.	30009488	2018	Adjuvant effect of a combination of Z and G, resulting in the expansions of Vγ9$\gamma\delta$T and Vα24NKT cells upon induction of antigen- specific CD8+ T cells using IFN DCs/P+Z+G. This indicates that het- erogeneous activation of lymphocytes shapes the responses to vaccination. These findings can help designing dendritic cell therapies combined with immunocheckpoint blockades.

Predictors of Response to Autologous Dendritic Cell Therapy in Glioblastoma Multiforme.	Jan CI et al.	Front Immunol	29910795	2018	In patients receiving postsurgical adjuvant immunotherapy with autologous dendritic cell/tumor antigen vaccine (ADCTA) in conjunction with conventional treatment of concomitant chemoradiotherapy (CCRT) with temozolomide, a lower PD-1+/CD8+ ratio is correlated with better overall survival and progression-free survival.
First results on survival from a large Phase 3 clinical trial of an autologous dendritic cell vaccine in newly diagnosed glioblastoma.	Liau LM et al.	J Transl Med	29843811	2018	The addition of DCVax-L autologous dendritic cell vaccine to surgical resection followed by 6 weeks of daily radiotherapy with concurrent temozolomide, then monthly temozolomide, has a benign safety profile and shows increased length of survival
Heat shock protein peptide complex-96 vaccination for newly diagnosed glioblastoma: a phase I, single-arm trial.	Ji N et al.	JCI Insight.	29769450	2018	Single arm controlled trial using HSPPC-96 vaccination (combined with standard Stupp regimen of radiotherapy plus concomitant and adjuvant temozolomide), which stimulated tumor-specific immune response, resulting in longer overall survival and higher progression-free survival rates.
IL-21 Increases the Reactivity of Allogeneic Human Vγ9Vδ2 T Cells Against Primary Glioblastoma Tumors.	Joalland N et al.	J Immunother	29683891	2018	Ex vivo sensitizations of Vγ9Vδ2 T cells by IL-21 showed that through their cytolytic activity, they significantly eliminated GMB tumor cells after orthotopic administrations in vivo.
Survival gain in glioblastoma patients treated with dendritic cell immunotherapy is associated with increased NK but not CD8+ T cell activation in the presence of adjuvant temozolomide.	Pellegatta S et al.	Oncoimmunology.	29632727	2018	Two-stage phase II study, glioblastoma patients were treated with dendritic cell immunotherapy and radiochemotherapy with temozolomide (TMZ) followed by adjuvant TMZ. DC vaccination induced a significant, persistent activation of NK cells, whose increased response was significantly associated with prolonged survival.
Dendritic Cells Enhance Polyfunctionality of Adoptively Transferred T	Reap EA et al.	Cancer Res	29093005	2018	CMV-ATCT-DC administered to GBM patients increased polyfunctional CMV-specific CD8+ T cells, correlating with overall survival (cannot confirm it is causally related). Polyfunctional T-cell responses as a potential biomarker for effective antitumor immunotherapy.

Cells That Target Cytomegalovirus in Glioblastoma.					
Efficacy of depatuxizumab mafodotin (ABT-414) monotherapy in patients with EGFR-amplified, recurrent glioblastoma: results from a multi-center, international study.	Van den Bent M et al.	Cancer Chemother Pharmacol.	29075855	2017	Depatuxizumab mafodotin monotherapy in EGFR amplified recurrent GBM patients increased OS and PFS, with tolerable ocular related side effects.
Phase I study of sorafenib and tipifarnib for recurrent glioblastoma: NABTC 05-02.	Nghiemphu PL et al.	J Neurooncol	28988377	2018	Combination of tipifarnib and sorafenib was toxic even at low doses, and even in patients with low toxicity, no further analysis was performed due to low OS and PFS.
Large Amino Acid Transporter 1 Selective Liposomes of l-DOPA Functionalized Amphiphile for Combating Glioblastoma.	Bhunia S et al.	Mol Pharm	28958145	2017	LAT1 mediated targeted chemotherapy with WP1066-loaded liposomes of Amphi-DOPA combined with in vivo DC-targeted DNA vaccination using a surviving encoded DNA vaccine enhanced overall survival in mice of >300% (compared to the untreated mice)
Co-delivery of tumor-derived exosomes with alpha-galactosylceramide on dendritic cell-based immunotherapy for glioblastoma.	Liu H et al.	Cancer Letters	28947140	2017	Co-delivery of tumor-derived exosomes with α-GalCer on a DC-based vaccine showed powerful effects in glioblastoma immunotherapy. This vaccine induced strong activation and proliferation of tumor-specific cytotoxic T lymphocytes, synergistically breaking the immune tolerance and improving the immunosuppressive environment.
Rindopepimut with temozolomide for patients with newly diagnosed, EGFRvIII-expressing glioblastoma (ACT IV): a randomised, double-	Weller M et al.	Lancet Oncol	28844499	2017	No significant difference in overall survival for the minimal residual disease when adding rindopepimut to standard chemotherapy with temozolomide

blind, international phase 3 trial.					
A phase II trial of autologous dendritic cell vaccination and radiochemotherapy following fluorescence-guided surgery in newly diagnosed glioblastoma patients.	Inogés S et al.	J Transl Med	28499389	2017	The use of tumor lysate-pulsed autologous DCs following surgery, radiotherapy and chemotherapy is safe. Will need further trial to confirm OS and PFS rates.
Sensitization of glioblastoma tumor micro-environment to chemo- and immunotherapy by Galectin-1 intranasal knockdown strategy.	Van Woensel M, et al	Sci Rep	28450700	2017	Nose-to-brain delivery of chitosan coated lipid nanocapsules modulates GBM microenvironment by suppressing Gal-1 expression, and sensitizes it to TMZ treatment, increasing long term survival.
Combination epidermal growth factor receptor variant III peptide-pulsed dendritic cell vaccine with miR-326 results in enhanced killing on EGFRvIII-positive cells.	Li J et al.	Oncotarget	28412740	2017	PEPvIII-DC-CTLs could specifically kill U87-EGFRvIII cells. miR-326 inhibited the expression and external secretion of TGF-β1 via the SMO/Gli2 pathway, reversing the immunosuppressive environment. Therefore DC-based vaccine combination with miR-326 can provide better outcomes of immunotherapy
Long-term Survival in Glioblastoma with Cytomegalovirus pp65-Targeted Vaccination.	Batich KA et al.	Clin Cancer Res	28411277	2017	CMV DC vaccines combined to dose intensified TMZ, can increase pp65 immunity leading to prolonged PFS and OS. 4 of 11 patients who received at least 3 vaccinations were progression-free at 59–64 months following surgery.
Autologous Heat Shock Protein Peptide Vaccination for Newly Diagnosed Glioblastoma: Impact of Peripheral PD-L1 Expression on Response to Therapy.	Bloch O et al.	Clin Cancer Res	28193626	2017	Vaccination with autologous tumor-derived heat shock protein peptide complexes in combination with standard therapy may improve survival, but warrants further studies. Also, patients with the most elevated expression of PD-L1 on circulating myeloid cells had the least response to vaccination.

Combating Established Mouse Glioblastoma through Nicotinylated-Liposomes-Mediated Targeted Chemotherapy in Combination with Dendritic-Cell-Based Genetic Immunization.	Saha S et al	Advanced biosystems	32646187	2017	IV administration of (WP-1066)-loaded liposomes of nicotinylated amphiphiles in combination with DC targeted immunization with tyrosinase-related protein-2 encoded DNA vaccine markedly enhances the OS of orthotopic glioblastoma-bearing mice (by >500% compared to the OS for the control group).
Syndecan-4 as a biomarker to predict clinical outcome for glioblastoma multiforme treated with WT1 peptide vaccine.	Takashima S et al.	Future science OA	28116121	2016	Low expression of SDC-4 in peripheral blood mononuclear cells prior to WT1 vaccination correlated with favorable clinical effects of WT1 peptide vaccination of GBM patients and therefore was a prognostic factor for long-term survivors.
α-type-1 polarized dendritic cell-based vaccination in recurrent high-grade glioma: a phase I clinical trial	Y. Akiyama et al	BMC Cancer	23270484	2012	α-type-1 polarizing DCs on GBM patients with mainly HLA-A*2402. Nine patients with high-grade glioma (1 case of HLA-A*0201, 8 of A*2402) were enrolled into a phase I study and given HLA-A2 or A24-peptide cocktail-pulsed mature DCs, which produced a large amount of IL-12. All 76 DC injections were safely administered to patients. These results suggested that peptide cocktail- treated α-type-1 DC-based immunotherapy was feasible, and had potential as a therapeutic tool against HLA-A24+ high-grade glioma.
Integration of autologous dendritic cell-based immunotherapy in the standard of care treatment for patients with newly diagnosed glioblastoma: results of the HGG-2006 phase I/II trial	H. Ardon et al	Cancer Immunol Immunother	22527250	2012	Full integration of autologous DC-based tumor vaccination into standard postoperative radioche- motherapy for newly diagnosed glioblastoma seems safe and possibly beneficial. These results were used to power the currently running phase IIb randomized clinical trial.

Heat-shock protein peptide complex- 96 vaccination for recurrent glioblastoma: a phase II, single-arm trial	O.Bloch et al	Neuro-Oncology	24335700	2013	The HSPPC-96 vaccine is safe and warrants further study of efficacy for the treatment of recurrent GBM. Significant pretreatment lymphopenia may impact outcomes of immunotherapy and deserves additional investigation.
Targeting EGF receptor variant III: tumor-specific peptide vaccination for malignant gliomas	C.A. Del Vecchio et al	Expert Reviews	22309662	2012	EGFRvIII has been identified as a tumor-specific oncoprotein expressed in a high percentage of GBM. Owing to the unique junction produced at the exon 1 to exon 8 boundary, this EGF receptor variant can be selectively targeted without affecting expression or function of the wild-type EGF receptor. Peptide vaccination using CDX-110, a 14-mer peptide against the junction, is currently being evaluated in Phase II/III clinical trials. Preclinical and clinical data demonstrate that CDX-110 is both safe and effective in evoking humoral and cellular immunity, and is correlated with apparent increases in median time to tumor progression and overall survival compared with historical controls as reviewed above.
Exploiting the glioblastoma peptidome to discover novel tumour-associated antigens for immunotherapy	V. Dutoit et al	Brain: a journal of neurology	22418738	2012	9 of the 10 peptides identified in GBM (one was omitted due to poor solubility) are now being developed in a multipeptide therapeutic vaccine designated IMA950. Recently, two phase I studies in first-line patients with GBM have started in the UK and USA (clinicaltrials.gov identifier codes NCT01403285 and NCT01222221). Immunogenicity of the vaccine can be assessed and set the base to improve immunization strategies by combining multipeptide vaccines with the most efficient immunomodulator.
Immunotherapy of rat glioma without accumulation of CD4+CD25+FOXP3+ regulatory T cells	E.Feng et al	Neural Regeneration research	25657686	2017	Co-immunotherapy, which may be due, in part, to inhibition of the accumulation of CD4+CD25+FOXP3+ Tregs. Unique method of using immunotherapy to treat glioma and has significant clinical potential. Several obstacles however need to be addressed before this therapy can be applied to humans with gliomas.
Rindopepimut: anti-EGFRvIII peptide vaccine, oncolytic	P.C. Gedeon et al	Drugs Future	25663738	2015	Rindopepimut is a promising new therapeutic agent for the treatment of EGFRvIII- expressing gliomas. Vaccinating patients with EGFRvIII-positive GBM is safe, well tolerated and produces immunity against EGFRvIII-expressing cells. More specifically, peripheral administration of rindopepimut/ GM-CSF results in the production of EGFRvIII- specific humoral and DTH responses. In patients with GBM, vaccination leads to the elimination of EGFRvIII-positive GBM cells without any associated autoimmunity. The ongoing phase III clinical trial (ACT IV) is necessary to continue to assess the safety and efficacy of rindopepimut/GM-CSF therapy for newly diagnosed GBM. There is a

					strong likelihood that rindopepimut vaccination for patients with EGFRvIII-positive newly diagnosed GBM will show sufficient safety and efficacy in the phase III randomized trial leading to approval by U.S. and European regulatory agencies.
Vaccination with Irradiated Tumor Cells Pulsed with an Adjuvant That Stimulates NKT Cells Is an Effective Treatment for Glioma	M.K. Hunn et al	Clinical Cancer Research	23147997	2012	Injection of irradiated tumor cells loaded with a-GalCer is a simple procedure that could provide effective immunotherapy for patients with high-grade glioma. -GalCer has been shown to have significant antitumor effects when injected intravenously as a sole agent.It has been found that the iNKT/DC axis has some deficiencies in patients. A mild impairment of iNKT cell expansion might be explained by corticosteroid treatment. We also observed reduced numbers of circulating myeloid dendritic cells but circulating CD14þ monocytes, known to be capable of differentiating into mature dendritic cells, were present in normal quantities and expressed normal levels of CD1d. Taken together, these data suggest there are no major defects in the iNKT/DC axis in patients with high-grade glioma and that a-GalCer–pulsed irradiated autologous glioma cells could be an effective vaccine in human patients.
Therapeutic vaccination against autologous cancer stem cells with mRNA-transfected dendritic cells in patients with glioblastoma	E. Osland Vik-Mo et al	Cancer Immunol Immunother	23817721	2013	mRNA transfected DC vaccine induced a glioma stem cell (GSC) -specific immune response without eliciting serious adverse reactions.. The use of sphere-forming capability for the propagation and enrichment of CSCs is well-established in the glioblastoma. The technology for enriching such cells is transferable to a variety of tumors; therefore, the immu- notherapy protocol presented here may be used as a model for targeting CSCs in other solid tumors.
Identification of Novel Human Leukocyte Antigen-A*0201-Restricted, Cytotoxic T Lymphocyte Epitopes on CD133 for Cancer Stem Cell Immunotherapy	J. Ji et al	Stem Cells Translational Medicine	24375541	2013	HLA-A*0201-restricted CD133 epitopes, CD133- 405 and CD133-753 identified. Unlike other potential CD133 epitopes examined, these two epitopes were strongly immunogenic in PBMCs from healthy donors and also naturally presented by GBM CSCs lines. Furthermore, studies in HLA-A*0201 expressing mice showed that these epitopes are indeed immunogenic in vivo but do not lead to autoimmune responses. Taken together, these results support CD133 as a suitable CTL target for GBM CSC immunotherapy. Based on these findings, a phase I trial of a dendritic cell vaccine using these CTL epitopes in recurrent glioblastoma has been initiated.

Intrinsically de-sialylated CD103+ CD8 T cells mediate beneficial anti-glioma immune responses	E. Jouanneau et al	Cancer Immunol Immunother	24893855	2014	De-sialylation of CD8 is required for hyper-responsiveness and beneficial anti-glioma activity by CD8 T cells. Because CD8 de-sialylation can be induced with exogenous enzymes (and appears particularly scarce on human t cells), it represents a promising target for clinical glioma vaccine improvement.
CD137 stimulation and p38 MAPK inhibition improve reactivity in an in vitro model of glioblastoma immunotherapy	C.Kuhnol , M et al	Cancer Immunol Immunother	24129764	2013	Study suggests p38 MaPK inhi- bition and anti-CD137 antibodies can enhance the immune response against GBM cells.
Varicella Zoster Virus Infection of Malignant Glioma Cell Cultures: A New Candidate for Oncolytic Virotherapy?	H. Leske et al	Anti-cancer Research	22493342	2012	Varicella zoster virus (VZV) exhibits an intrinsic oncolytic potential in malignant glioma cell cultures and might be a novel candidate for virotherapy in GBM.
Mouse glioma immunotherapy mediated by A2B5+ GL261 cell lysate-pulsed dendritic cells	M. Xu et al	Journal of Neurooncology	24398615	2014	Vaccination with A2B5+ GL261 cell lysate-pulsed DCs only has some glioma preventive effect. In order to obtain therapeutic effect, DC vaccination requires reduction of tumor load (tumor resection) as much as possible and elimination of glioma-induced immune suppression. Increased effectiveness of DC is noted by also inhibiting transforming growth factor-beta or depletion of Tregs.
Phase I trial of a multi-epitope-pulsed dendritic cell vaccine for patients with newly diagnosed glioblastoma	S. Phuphanich et al	Cancer Immunol Immunother	22847020	2012	Phase I study of ICT-107 demonstrated the feasibility, safety, and bioactivity of a TAA-pulsed DC vaccine GBM patiens. Expression of AIM2 and MAGE1 antigens in the pre-vaccine tumors correlated with prolonged survival as measured by PFS and OS in ND-GBM patients, while expression of HER2 and gp100 showed a trend toward longer PFS and OS.
Human umbilical vein endothelial cell vaccine therapy in patients with recurrent glioblastoma	M. Tanaka et al	Cancer Science	23106822	2012	HUVEC vaccine therapy is a promising new treatment modality, without important adverse effects. Acquisition of resistance to multi-target immunotherapy, namely the HUVEC vaccine therapy, seems to be less frequent. As a matter of course, a large-scale prospective study will help confirm our present results.
Live attenuated measles virus vaccine therapy for locally established malignant glioblastoma tumor cells	A.M. Al-Shammari et al	Oncolytic Virotherapy	n/a	2014	Measles virus (MV) vaccine strains replicate efficiently in GBM tumor cells as well as in Vero and RD cells and can be further investigated for future use as GBM recommended therapy.

Autologous T-cell Therapy for Cytomegalovirus as a Consolidative Treatment for Recurrent Glioblastoma	A. Shuessler et al	Cancer Research	24795429	2014	Adoptive immunotherapy of patients with recurrent GBM with CMV-specific T cells is safe and may provide long-term clinical benefits. Adoptive T-cell immunotherapy may be used both therapeutic and prophylactic settings. CMV-specific T-cell–based immunotherapy should be considered as a consolidative treatment following primary diagnosis of GBM for the prevention of recurrent disease.
